# Genome-Wide Mapping of Collier *In Vivo* Binding Sites Highlights Its Hierarchical Position in Different Transcription Regulatory Networks

**DOI:** 10.1371/journal.pone.0133387

**Published:** 2015-07-23

**Authors:** Mathilde de Taffin, Yannick Carrier, Laurence Dubois, Laetitia Bataillé, Anaïs Painset, Stéphanie Le Gras, Bernard Jost, Michèle Crozatier, Alain Vincent

**Affiliations:** 1 Centre de Biologie du Développement, UMR 5547 CNRS Université de Toulouse 3, 118 route de Narbonne, F-31062, Toulouse cedex 09, France; 2 Plate-forme bio-informatique Genotoul/MIA-T, INRA, Borde Rouge, 31326, Castanet-Tolosan, France; 3 Institut de Génétique et de Biologie Moléculaire et Cellulaire, CNRS/INSERM/Université de Strasbourg, 67404, Illkirch, France; Simon Fraser University, CANADA

## Abstract

Collier, the single *Drosophila* COE (Collier/EBF/Olf-1) transcription factor, is required in several developmental processes, including head patterning and specification of muscle and neuron identity during embryogenesis. To identify direct Collier (Col) targets in different cell types, we used ChIP-seq to map Col binding sites throughout the genome, at mid-embryogenesis. *In vivo* Col binding peaks were associated to 415 potential direct target genes. Gene Ontology analysis revealed a strong enrichment in proteins with DNA binding and/or transcription-regulatory properties. Characterization of a selection of candidates, using transgenic CRM-reporter assays, identified direct Col targets in dorso-lateral somatic muscles and specific neuron types in the central nervous system. These data brought new evidence that Col direct control of the expression of the transcription regulators *apterous* and *eyes-absent* (*eya*) is critical to specifying neuronal identities. They also showed that cross-regulation between *col *and *eya* in muscle progenitor cells is required for specification of muscle identity, revealing a new parallel between the myogenic regulatory networks operating in *Drosophila* and vertebrates. Col regulation of *eya*, both in specific muscle and neuronal lineages, may illustrate one mechanism behind the evolutionary diversification of Col biological roles.

## Introduction

Differential gene expression underlying animal development and cell differentiation is mediated at the transcriptional level by Cis-Regulatory Modules (CRMs), which contain short DNA motifs acting as binding sites for sequence-specific transcription factors (TFs) [1]; [2]. Increasing organismal complexity throughout metazoan evolution has been paralleled by the expansion of TF families, allowing sub-specialization of each family member, *via* changes in either expression pattern or/and biochemical properties. One peculiar situation is the COE (Collier/EBF/ Olf-1) family of sequence-specific TFs, which display a HLH dimerization motif associated with a specific DNA-binding domain [3]; [4]; [5]. The COE family does comprise a single member in all invertebrates, from sponges to ascidians [6]; [7]; [8], and 4 members (Early B Cell Factor; EBF1-4) in vertebrates [9]; [10], indicating that *coe* gene duplications only occurred at the origin of vertebrates [7]. Pioneering studies showed that EBF binds DNA *in vitro* as dimer, to a consensus palindromic sequence ATTCCCNNGGGAAT [11]; [12]. The high degree of primary sequence conservation and lack of expansion of COE proteins contrast with their diversity of functions, as revealed by analyses of mutants, both in vertebrates [13]; [14], [15]; [16]; [17]; [18], nematodes [19]; [20]; [21] and *Drosophila* ([22] and references in the text below).

*Drosophila* Collier (Col) (Flybase; Knot (Kn)) in involved in multiple developmental programs in embryos: early head patterning; specification of muscle progenitor cells (PCs) and founder cells (FCs) at the origin of dorso-lateral somatic muscles; specification of lymph gland (LG) cells, the larval hematopoietic organ; control of neuron identities in both the peripheral and central nervous system [5]; [23]; [24]; [25]; [26]; [27]; [28]; [29]; [30]; [31]; [32]. Yet, despite a wealth of genetic and developmental studies, only two direct Col targets, *hh* and *col* itself, have been characterized so far [33]; [34].

To get deeper insight into Col regulatory roles in different developmental processes, we sought to identify direct Col target genes. Here, we used ChIP-seq to perform a genome-wide analysis of Col binding to chromatin at mid-embryogenesis, (stages 13–14), a time frame when Col is expressed in several cell types in the mesoderm and nervous system. This analysis identified 415 potential direct Col target genes. Among those, 64 encode transcription regulators, including several sequence-specific TFs previously shown genetically to act downstream of Col in the head, specific somatic muscles and neuronal lineages, thereby validating our approach. More detailed analysis of a selection of targets, and corresponding CRMs, showed that Col directly regulates the expression of *apterous* (*ap*), *eyes absent* (*eya*), *nerfin-1* and, very likely, *even-skipped* (*eve*), in specific neuronal lineages, thus contributing, both directly and via the direct regulation of other TFs, to transcriptional codes specifying different neuron identities. It also revealed that cross-regulation between *eya* and *col*, in somatic muscle progenitors, is required for specification of muscle identity. Col binding peaks in numerous other TFs offers as many new entries to investigate the combinatorial control of cell identity.

## Materials and Methods

### Chromatin immunoprecipitation and sequencing

ChIP experiments were performed according to [35], using stage 13–14 *col* 2.6_0.9^ColCONS^ *-lacZ* embryos, with a mix of three different monoclonal antibodies recognizing separate epitopes of the Col protein. The mock was monoclonal HA antibody (HA.11 Clone 16B13-Covance, Dedham, Massachusetts, USA). To improve the purification yield, we used a competitive elution with purified recombinant Col protein (Kn-RB isoform; http://flybase.org); this step contaminated the ChIP-DNA with plasmid DNA, requiring bio-informatics elimination of the contaminating sequences. The precipitated DNA was quantified using Qubit dsDNA H Assay Kits (invitrogen#Q32851). Real time quantitative PCR was performed on a MyiQ single color real time PCR detection system (Biorad). CT values were collected and analysis was performed using the 2(-Delta Delta C(T)) method [36], using *cg11964* to normalize calculations of relative expression. For comparison, we substituted the consensus EBF/Col binding motif established *in vitro* (TCCCNNGGGA; [11] for the endogenous TGTCNNGGGA site in the reporter construct *col* 2.6_0.9^ColCONS^ *-lacZ*. Primers sequences for *col* and *col* 2.6_0.9^ColCONS^ are available on request. All qRT-PCR data are representative of three independent experiments. DNA from two independent Col and Mock iPs was pooled before ChiP fragment amplification and High-throughput sequencing (Genome analyzer IIx, Illumina; Microarray and sequencing platform, IGBMC, Illkirch). Reads were aligned to the *D*. *melanogaster* genome (BDGP5) using Bowtie v0.12.7 [37]. Data are available at Gene expression Omnibus, Accession number GSE67805.

### Identification of Col binding peaks

Col peaks were searched using SISSRs v1.4 [38]; [39], run with default parameters except for the following options: pValue threshold = 0.1, eValue threshold = 1500, one read per genomic coordinate, average fragment length = 191. 559 Col binding peaks retrieved by SISSRs were further analysed.

### *de novo* motif discovery

MEME-ChIP v4.10.0 [40] was used to search 200 bp of DNA, centred on the summit of each of the 559 Col peaks. Motif discovery was performed by scanning both DNA strands for 4 to 25 nucleotides long motifs, with a distribution probability of zero or one occurrence per sequence and a 4-order Markov model as background reference. Results from SISSRS and MEME analyses are provided as S1 File and dataset GSE 67805.

### Genome annotation and GO enrichment of Col binding peaks

Peak Analyzer [41] was used to associate each Col ChiP-Seq peaks with one gene in the *Drosophila* genome (BDGP 5.74). Peaks were associated with the overlapping gene when in introns or the nearest gene transcription start. Enrichment of Col target genes for GO biological processes was using GeneCodis (http://genecodis.cnb.csic.es/)

### Reporter constructs and transgenic lines

pDEST-moeGFP and pDEST-moeRFP were made by replacing Gal4 sequences from pbGUw (addgene plasmid #17575) by moeGFP and moeRFP sequences, respectively. For candidate Col targets (Table 1), 1kb long DNA fragments centered on the Col binding site were cloned upstream of *moeGFP* by Gateway recombination (invitrogen–life technologies) into *pDEST-moeGFP*. Site-directed mutagenesis of the Col binding site(s) (S2 Table) was done by PCR. The ap_Col^mut^ and eya_Col^mut.1^ et eya_Col^mut.2^ were made in pDEST-moeRFP. All moe-GFP/RFP reporters were inserted at position 68A4 on the third chromosome by injection into *nos-phiC31-NLS* and made homozygous. Other fly stocks are available at the Bloomington Stock Center.

**Table 1:** Candidate Col direct targets and linked CRMs.

Candidate gene	Interpro domains (http://flybase.org/)	Col overlapping expression	Number of peaks	CRM	Peak height	CRM/Col overlapping expression	Effect of mutating the Col binding site
***Ama***	Immunoglobulin-likedomain	Hypopharyngeallobe, mesoderm	1	*Ama*_Col	2.53	Hypopharyngeal lobe	loss
***ap***	Homeobox domainZinc Finger, LIM-type	Ventral nervecord	1	*ap*_Col	6.08	dAp neurons Tv neurons	Loss
***cnc***	Basic leucine Zipper(bZIP) domain	Hypopharyngeallobe	1	*cnc*_Col	8.03	Hypopharyngeallobe	Down-regulation
***eya***	Protein tyrosinephosphatase activity	MesodermVentral nervecord	2	*eya*_Col	3.99/ 2.39	dAp neurons/Tv neuronsDA3 muscle	Loss/Down-regulationLoss
***jing***	Zinc finger C2H2	mesoderm	5	*jing*_Col	5.17	Lymph gland	Down-regulation
***Mrtf***	SAP domain	Somaticmuscles	1	*Mrtf*_Col	2.57	DA3 muscle	No change
***nerfin-1***	Zinc finger C2H2	Ventral nervecord	1	*nerfin-1*_Col	5.59	Subset of CNSneurons	Loss
***Oaz***	Zinc finger C2H2	n.d.	6	*Oaz*_Col	5.12	DA3 and DT1muscles	Loss
***so***	Homeobox domain	Trunkmesoderm	1	*so*_Col	5.32	DA3 and LL1muscles	No change
***tkv***	Serine/Threonineprotein kinase, TGFBreceptor	Trunkmesoderm	1	*tkv*_Col	4.24	Lymph gland	Down-regulation
***col***	Transcription FactorCOE	+	1	*col*2.3–0.9	3.26	Hypopharyngeal lobe/DA3 muscle	No change/Down-regulation

n.d.: not detected

### Immunohistochemistry and *in situ* hybridization

Immunostaining and *in situ* hybridization procedures were as in [33]. The following primary antibodies were used: mouse anti-β-galactosidase (Promega) 1/800; anti-Col 1/50; rabbit anti-GFP (Torrey) 1/500; anti-RFP (Rockland 1/500); anti-Nau 1/100 (B. Paterson, Bethesda, USA), anti-β3-Tubulin 1/5000 (R. Renkawitz-Pohl, Marburg, Germany). Secondary antibodies were Alexa Fluor -488, -647, -555 conjugated antibodies (1:300; Molecular Probes). Mounting samples for confocal microscopy (Leica SP2, SP5 and SPE microscopes, Wetzlar, Germany; 20x and 63x objectives) was in Vectashield medium (Vector Laboratories). The Vectastain ABC Kit PK-401, from Vector, was used for DAB (3, 3’-diaminobenzidine) immunostaining; phosphatase-conjugated antibodies for BCIP/NBT detection of ISH transcripts were from Roche. DAB immunostaining experiments were repeated at least 3 times and NBT/BCIP ISH at least twice with large collections of embryos. At least 10 randomly selected embryos at one given developmental stage were recorded for each experiment, using a 20x objective. Effect of mutating the Col binding site(s) was considered as significant when >80% of embryos showed a change in expression pattern between the intact and mutated reporter constructs, except when otherwise indicated in the text. Images shown in figures and supporting figures are representative examples. Colocalisation of signals from 2 different fluorochromes used Image J colocalization highlighter plugin (Pierre Bourdoncle, Institut Cochin, Paris, France).

## Results

### Genome-wide mapping of Col binding sites to chromatin in stage 13–14 embryos

**Fig 1:**
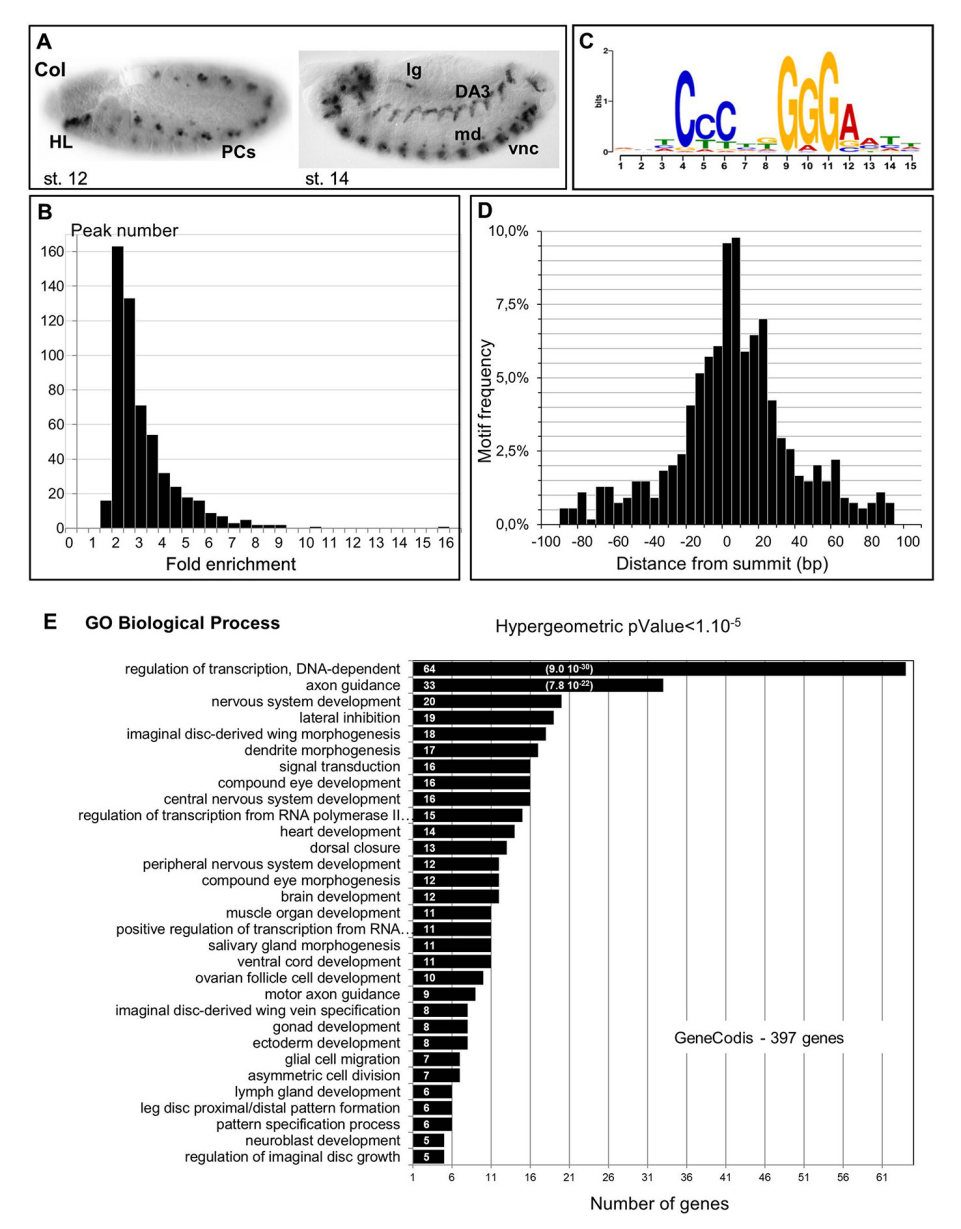
Genome-wide mapping of Col binding sites. (A) Col expression in stage 12 and stage 14 embryos, lateral view. In this and all subsequent figures, embryos are oriented anterior to the left. HL: hypopharyngeal lobe; PCs: dorso-lateral muscle progenitors; vnc: ventral nerve chord; lg: lymph gland; md: class IV multidendritic neuron. (B) Fold enrichment distribution of the 559 Col ChIP peaks selected using SISSRs. (C) Single most enriched sequence motif identified by MEME analysis in the 559 Col binding peaks. (D) Graphical representation of the position of Col-binding motifs relative to the center of Col binding peaks. The axis gives the number of motifs in each cluster. (E) GO clusters enriched in putative Col direct target genes. The p-Value of the top two clusters is given in brackets.

In order to identify Col direct targets, we used chromatin from 10 to 12h old *Drosophila* embryos (stages 13–14). During stages 11–13, Col is expressed the muscle PCs and FCs at the origin of the dorso-lateral (DL)—DA3, DO3, DO4, DO5, LL1 and DT1- somatic muscles, and starts to be expressed in the ventral nerve cord (VNC); it is also expressed in the hypopharyngeal lobe (HL) (Fig 1A). At stage 14 and later, it is expressed in the differentiating DA3 muscle, about 50 VNC neurons and 2 or 3 multidendritic (md) neurons per hemisegment, and the developing LG [5]; [24]; [25]; [31] (Fig 1A). To immuno-precipitate Col-bound chromatin fragments, we opted for a mix of monoclonal anti-Col antibodies. As an internal control for IP specificity, we used a reporter transgene carrying a modified *col* CRM, *col* 2.6_0.9^ColCONS^, whose activity in the DA3 muscle depends upon direct Col binding [33]. Quantitative real time PCR [36] of DNA fragments covering the endogenous and transgenic *col* CRMs was performed on DNA samples from two independent IPs. It showed a significant enrichment of both fragments, of around 2 (2.08 and 2.19) and 4 (3.90 and 4.26) folds, respectively, compared to the intergenic region of *cg11964*, a control housekeeping gene [35]. This differential enrichment both confirmed the efficiency of Col antibodies and indicated that the nucleotide sequence of the Col binding motif could influence the occurrence and/or stability of contextual *in vivo* Col binding.

Low amounts of chromatin were obtained for each IP, correlating well with the small number of Col-expressing cells. We therefore pooled IP samples before sequencing. 18.3x10^6^ and 14.6x10^6^ immuno-precipitated fragments of 190 bp average size were sequenced for the Col-IP and mock (HA-IP) samples, respectively (dataset GSE 67805). The sequences were aligned to the *D*. *melanogaster* genome (BDGP release 5) using Bowtie v0.12.7 [37]. 16.1x10^6^ and 13.1x10^6^ unique reads for the Col and mock-IP, corresponding to 25 and 22 times the *Drosophila* genome size, respectively, were kept for analysis. Peak calling using the SISSRs software [42] detected 559 Col binding peaks (pvalue threshold = 0.1, eValue threshold = 1500), with a fold enrichment ranging from 1.94 to 16.1 (Fig 1B and S1 Table). The peaks were located either in introns or intergenic regions, consistent with Col binding *in vivo* to cis-regulatory regions.

*de novo* motif discovery was then performed on the entire set of 559 Col peaks, using the MEME suite software [43]. 200 nucleotides long windows centered on each peak’s summit were considered for this analysis (S1 File). It revealed that 97% of the peaks (542/559) contain one motif of consensus sequence CCCnnGGGA (Fig 1C). This consensus site is similar to the consensus *in vivo* binding site determined for mouse EBF in cultured lymphomas [44]. Significant enrichment of positions 13 and 14 for A and T nucleotides, respectively, was also consistent with the ATTCCCNNGGGAAT sequence of the *in vitro* EBF binding site defined by selex [11]. The calculated E-value: 2,1e-381 and predominant position of the CCCnnGGGA motif close to the center of the ChIP peak (Fig 1D) supported the conclusion that this motif is bound by Col *in vivo*. MEME analysis failed, however, to reveal other significantly enriched motifs, which could have represented binding sites for other sequence-specific TFs acting synergistically with Col in the different cell types where Col is expressed.

### Direct Col-target genes are enriched in transcription factors

PeakAnalyzer [41] associated the 559 Col ChIP-seq peaks to 415 genes (dm3/FlyBase R5.74). Several peaks (between 2 and 9) were associated with the same gene in 95 cases (S1 Table). 150 peaks (27%) were located 5’ and 100 (18%), 3’ of the nearest transcription start site (TSS), while most other peaks (309; 55%) were located in introns. Of course, this association did not exclude that, in some cases, the Col-bound region could act as remote enhancer for other/and additional genes [45], especially for those peaks found further than 10 kb from the nearest TSS (72 peaks; 13%). In 43 cases, the automatic peak association could be manually curated, to account for gene association issued from compared expression of Vienna Tiles (VT) reporter constructs [45] or other CRMs described in REDFly3.3., with that of nearby genes. For 7 peaks, of which 4 were located far away from the nearest transcription start, this analysis modified the associated gene (S1 Table). The described embryonic expression patterns of 176 of the 415 Col targets (http://insitu.fruitfly.org/) indicated that 100 (57%) are expressed in the CNS and 45 (25%) in the mesoderm, consistent with the Col expression pattern in stage 12–14 embryos. In order to further associate Col target genes to biological processes we used GeneCodis [46]; [47]; [48] to perform a Gene Ontology (GO) analysis (Fig 1E). 397 out of 415 Col-bound genes had GO annotations for Biological Processes and were considered by GeneCodis. 156 were represented in at least one cluster (>5 genes) of p-value<10^−5^. Highly enriched GO terms identified several developmental processes, correlating with the diverse functions played by Col during mid-embryogenesis (Fig 1E). GO terms further revealed a statistically significant enrichment in the category “regulation of transcription”, with 64 of 397 (16%, P-value 9x10^-30^) annotated Col targets present in this category. This could, in part, be due to larger than average regulatory regions of developmental control genes [49]. Yet, several of these TF genes were already shown to act genetically downstream of Col in different cell types, thus validating our dataset. One example was *cap n’ collar* (*cnc*), which acts downstream of Col in head patterning [5]; [26]. In addition to *cnc*, the dataset included *pox-meso* (*poxm*), *slouch/S59* and *col* itself which are regulated by Col in specific muscle lineages [33]; [32], and *apterous* (*ap*), *even-skipped* (*eve*), and *eyes-absent* (*eya*) which are regulated by Col in specific subsets of VNC neurons [28]; [31]. *eve* expression depends upon Col in a specific subset of neurons, the Eve-lateral (EL) neurons [31]. Previous dissection of *eve* cis-regulatory elements identified a 0.7kb genomic region, EveEL, specifically required for *eve* expression in EL neurons [50]; [51]. We found that Col binding to *eve* precisely mapped within EveEL (S1 Fig). The congruence between *col*/*eve* epistatic interaction, and matching genomic positions of EveEL and *in vivo* Col binding, led us to propose that Col binding reflected direct *eve* regulation in EL neurons. By extension, GO analysis suggested that Col directly regulates the transcription of various other TFs.

In summary, our ChIP-seq analysis identified 415 potential direct Col targets, among which 64 transcription regulators, suggesting that Col occupies a hierarchical position in a diversity of transcription regulatory networks. More than half of putative Col targets are “unknown” genes for which Col binding provides an entry site for studying their developmental expression and biological role.

### Selection of candidate Col direct targets

Identification of Col chromatin binding sites provided an opportunity to identify genes regulated in different Col-expressing cells and the corresponding CRMs. Here, we focused on a small set of 10 candidate Col targets. Selection was based on a number of criteria, including a range of peak heights (from 2.39 to 8.03); genes displaying from 1 to 6 Col peaks; documented expression, especially in the nervous system and/or mesoderm (http://insitu.fruitfly.org/); GO analysis (Fig 1E) and previous literature on Col embryonic functions. It included 8 TFs or co-factors—*ap*, *eya*, *jing*, *Myocardin-related transcription factor* (*Mrtf*), *nervous fingers-1* (*nerfin-1*), *O/E-associated zinc finger protein* (*Oaz*), *and sine oculis* (*so*)—and two transmembrane proteins, *Amalgam* (*Ama*), and *thickveins* (*tkv*) (Table 1). Among TFs, *ap* and *eya* were chosen because of prior evidence for their regulation by Col in specific neurons, with no evidence that this control was direct [28]; [52]. *eya* encodes a protein with tyrosine phosphatase activity which is a partner of SIX homeodomain TFs (D-Six4, Optix and So in *Drosophila*) [53,54]. *so* is the only *SIX* gene bound by Col *in vivo*. Oaz is the *Drosophila* ortholog of vertebrate Oaz/Zfp243, the only TF reported to physically interact with EBF [55]; [56].

For each gene in our selection, we cloned 1 kb fragments centered on the Col peak summit, upstream of a *moe-GFP* reporter construct and followed GFP expression in transgenic *Drosophila* lines. Each CRM construct was given the name of the gene, followed by _Col (Table 1 and S2 Table). For *jing* and *Oaz* which display 5 and 4 Col peaks, respectively, we analyzed one peak per gene, where the nucleotide sequence of the predicted Col recognition site was well conserved between 12 different *Drosophila* species ([57]; [58]; Dataset GSE 67805). For *eya*, which displays 2 nearby peaks, they were analysed together as well as separately. To precisely determine the role of Col binding for CRM activity, we made a parallel series of constructs in which the predicted Col binding site(s) was mutated at 3 nucleotide positions by substituting CCCNNCCC for CCCNNGGG (S2 Table). This substitution was shown to abolish EBF binding *in vitro* [11]. As an *in vivo* control, we mutated the Col binding site in the *col*2.3–0.9 CRM (*col*2.3–0.9^mut^). *col-2*.*3–0*.*9* ^*mut*^ activation in the head was not modified, while expression was severely reduced in the DA3 muscle where *col*2.3–0.9 is subject to autoregulation [33], showing that our reporter strategy could identify context-dependent direct regulation by Col (S2 Fig).

**Fig 2:**
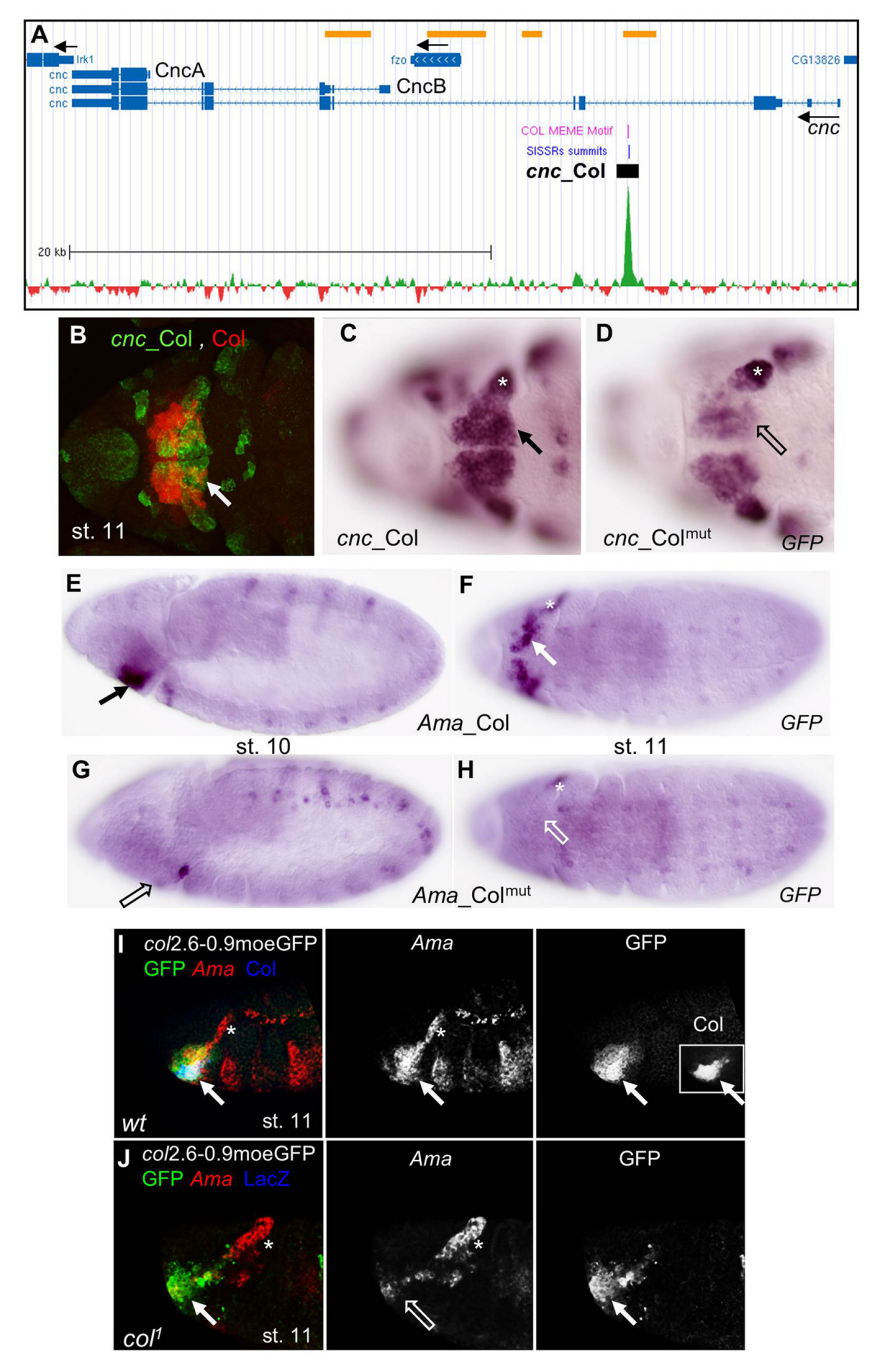
Col control of *cnc* and *Ama* expression in the head. (A) Annotation of the Col peak in *cnc*, adapted from *Gene Browser* (GEO submission GSE67805). 39,5 kb of *cnc* genomic region are shown (Chr3R: 19.009.000–19.048.500) with the Flybase gene annotation indicated by bars (transcribed regions) and intervening blue lines (introns). Black arrows indicate the direction of transcription of *cnc* and *fuzzy onions* (*fzo*), *inwardly rectifying potassium channel 1* (*Irk1*). The *cnc* transcripts coding for the protein isoforms CncA and CncB are indicated. ChIP-seq data for Col (green) substracted from HA (mock) data (red) are shown on the bottom. The Col Dam-ID binding regions [59] are indicated by yellow bars, top line. The summit of the ChIP-Col peak identified by SISSRs and position of the Col binding site(s) identified by MEME are indicated by blue and violet lines, respectively; the position of *cnc_*Col is represented by a black box; scale is indicated. (B-D) Ventral anterior views of stage 11 embryos. (B) Overlap between *cnc_*Col (GFP, green) and Col (red) expression in the HL (white arrow). (C) *cnc_*Col and (D) *cnc_*Col^mut^ mRNA expression, showing down-regulation of *cnc_*Col^mut^ in the mandibular segment (open arrow). (E,F) *Ama*_Col, (G,H) *Ama*_Col^mut^ expression in stage 10 (E,G) and 11 (F,H) embryos. HL *Ama*_Col expression (arrow) is lost in *Ama*_Col^mut^ (open arrow). (I, J) Overlap between *Ama* (red), Col (blue), and *col*2.6–0.9moeGFP (green) expression in the HL (white arrow) in stage 11 wt (I), and *col* ^*1*^ mutant embryos (J). Separate signals for *Ama*, GFP (I, J) and Col (inset in I, left panel) are shown in black and white. *Ama* expression is specifically lost in the HL in *col* ^*1*^ mutants. The asterisk in C, D, F, H, J indicates *Ama* and *cnc* expression independent on Col.

**Fig 3:**
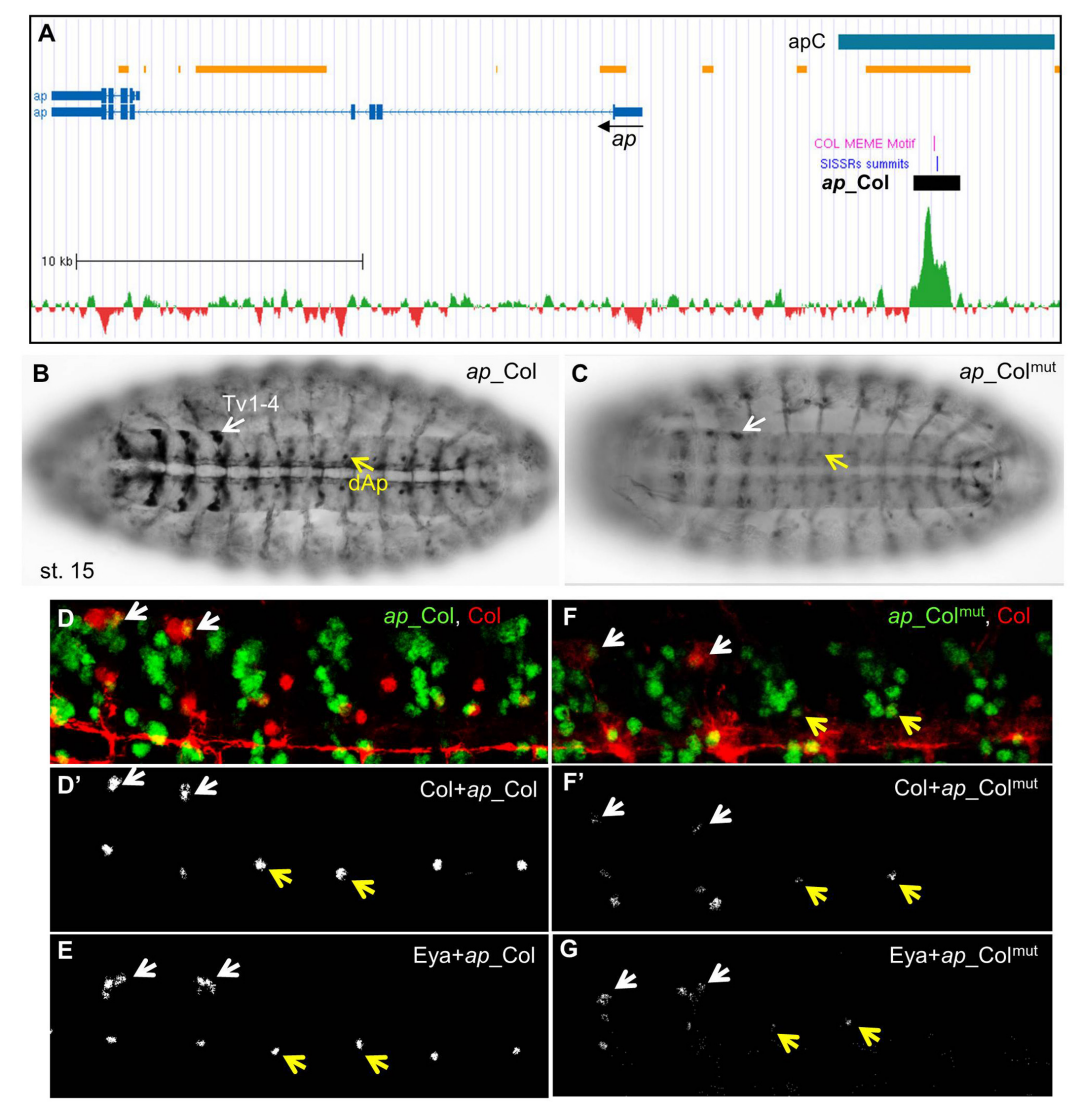
Col direct control of *ap* expression in Ap neurons. (A) Annotation of the Col peak in *ap*, same representation as in Fig 2A; 35.8 kb of the *ap* genomic region are shown (Chr2R: 1.593.000–1.628.800); the previously described apC enhancer is represented by a blue box. (B) *ap*_Col (GFP) expression in the dAp (yellow arrow) and Tv1-Tv4 neurons (white arrow) in stage 15 embryos, ventral view. (C) *ap*_Col^mut^ expression is severely reduced in dAP neurons and Tv neurons. (D,D’) Close up view of 4 segments of stage 16 embryos, showing the specific overlap between Col (red) and *ap*_Col (green) in the Tv1 and dAp neurons. (E) all Tv neurons express *ap*_Col and Eya. (F,G) *ap_*Col^mut^ expression is lost in dAp and strongly reduced in Tv neurons.

**Fig 4:**
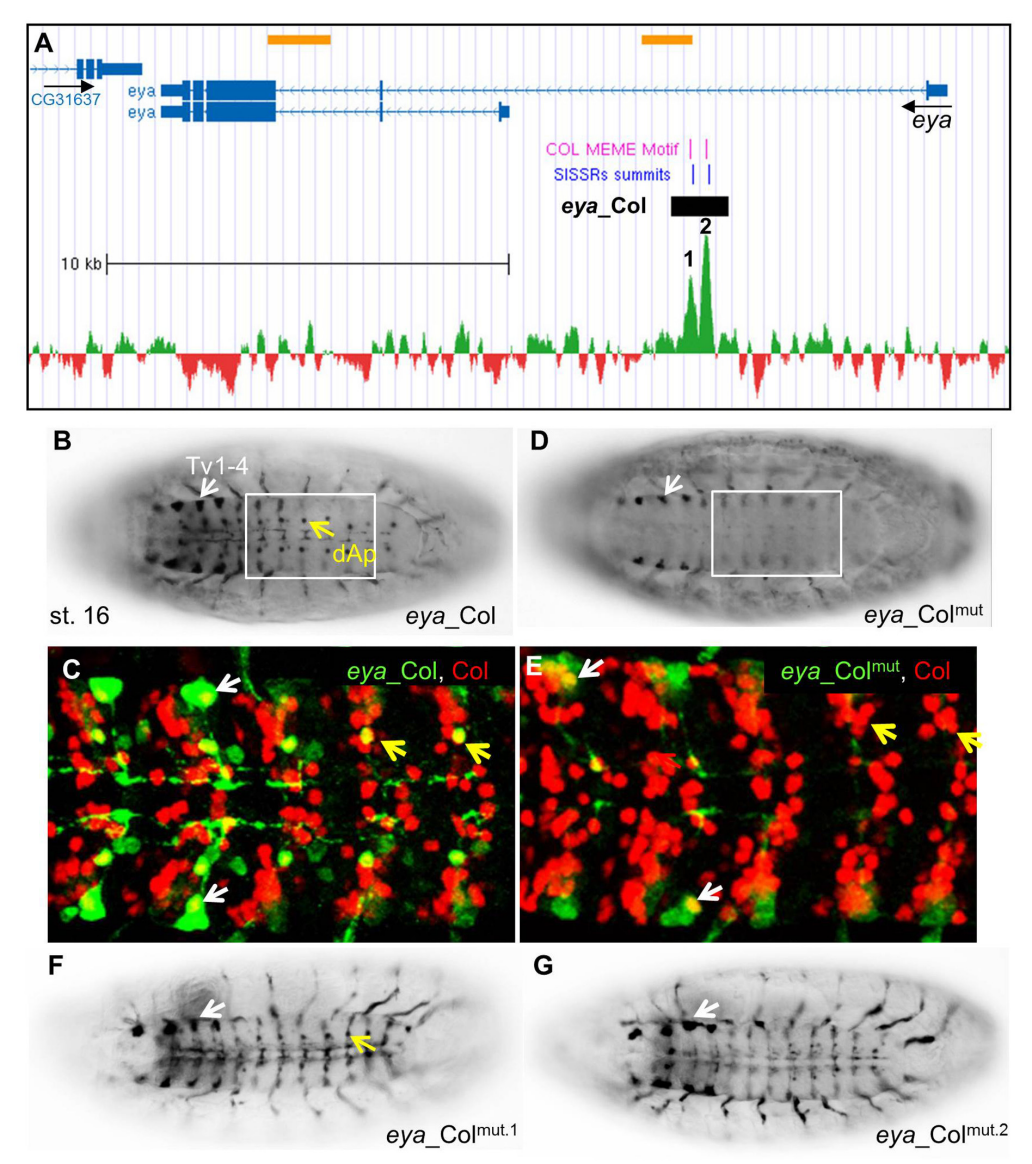
Col direct control of *eya* expression in Ap neurons. (A) Annotation of the Col peaks in *eya*, same representation as in Fig 2A; 24 kb of the *eya* genomic region are shown (Chr2L: 6.524.500–6.548.500); the summits of the two ChIP-Col peaks are numbered 1 and 2. (B, C) *eya_*Col (GFP) expression in the dAp (yellow arrow) and Tv1-Tv4 neurons (white arrow) in stage 16 embryos, ventral view. (C) Close up view of abdominal segments, showing the specific overlap between Col (red) and *eya_*Col (green) in the dAp (yellow arrow) and Tv1 neurons (white arrow). (D, E) *eya_*Col^mut^ expression is lost in dAP neurons and reduced in TV neurons. (F, G) Mutation of the Col binding site 2 (G), but not site 1 (F) eliminates *eya_*Col (RFP) expression in dAp neurons (yellow arrow).

**Fig 5:**
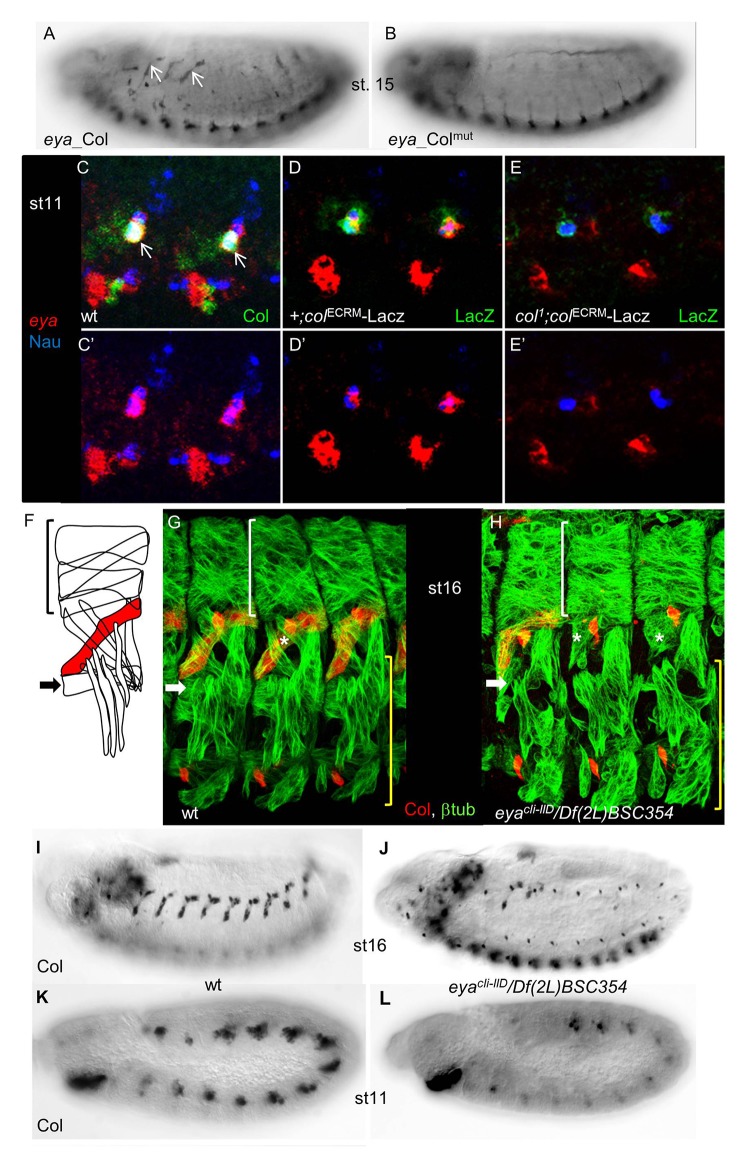
Cross-regulation between *eya* and *col* is required to specify dorso-lateral muscles. (A) *eya_*Col (GFP) expression in the DA3 muscle (arrows) in stage 15 embryos, is not detected for *eya_*Col^mut^ (B). (C-E) Triple staining of st11 wt (C), *+;col* ^*eCRM*^ *-lacZ* (D) and *col* ^*1*^ *;col* ^*eCRM*^ *-lacZ* (E) embryos for Nau (blue), *eya* transcripts (red), and either Col (C) or β-galactosidase (D,E) (green), shows co-expression of Col, *col* ^e-CRM^-LacZ, Nau and *eya* in DL muscle PCs (white arrow). (C’-E’) only Nau and *eya* stainings are shown. *eya* transcription is specifically lost in DL PCs in *col* ^*1*^ mutant embryos (E,E’); dorso-lateral view of the T2 and T3 segments is shown. (F) Schematic drawing of the dorsal, dorso-lateral and lateral transverse muscles in a stage 16 wt embryo, with the DA3 muscle in red and the LL1 muscle indicated by an arrow. (G, H) Staining of stage 16 wt (G) and (H), *eya* ^*cIi-IID*^ */Df(2L)BSC354* (null) mutant embryos for Col (red) and β3-tubuliin (green). Lateral view of 3 segments. In absence of *eya*, Col expression is lost in most segments, the LL1 muscle is missing (white arrow) and the DA3 muscle (asterisk) malformed. White brackets indicate dorsal, unaffected muscles while the lateral transverse and ventral muscles (yellow brackets) are moderately affected. (I-J) Col immunostaining of st.16 wt (I), and (J) *eya* mutant embryos, showing the loss of Col muscle expression in most segments (see also G, H), in *eya* mutants. (K, L) promuscular Col expression, early stage 11, is reduced in *eya* mutant embryos (L), compared to wt (K).

Analysis of the 10 candidate CRMs revealed overlap with Col expression in various tissues (Table 1), including the head hypopharyngeal lobe (HL) (*Ama*, *cnc*; Fig 2), the LG (*jing* and *tkv*, S3 Fig), specific VNC neurons (*ap*, *eya*, *nerfin-1;* Figs 3 and 4 and S4 Fig), body wall muscles (*eya*, *Mrtf*, *oaz*, *so*, Fig 5 and S5–S7 Figs). *tkv_*Col and *jing_*Col were expressed in numerous cell types, including the LG. Since LG development is a dynamic process [27], we compared *tkv_*Col with *tkv_*Col^mut^, and *jing_*Col with *jing_*Col^mut^ expression by ISH at two developmental stages, in order to detect subtle transcriptional regulation potentially hindered by the time lag between moeGFP transcription and protein accumulation (S3 Fig). Upon mutation of the Col binding site, expression of both reporters was specifically decreased in the LG, indicating that Col positively regulates *tkv_*Col and *jing_*Col activity in this tissue. However, since the decreased expression was only indubitable in about half of the embryos, these targets were not investigated in more detail.

### Col targets in the head

Col is required for head patterning in the embryo, downstream of gap genes [23], more specifically in parasegment 0 (posterior intercalary and anterior mandibular segment; also described as HL from stage 11), where it regulates expression of the segment polarity gene *hh* and the segment identity gene *cnc*. Other than *col* itself [33], *hh* was the only direct Col target previously characterized [34]. Col ChIP-seq failed to detect binding to *hh*, perhaps because it is regulated at earlier developmental stages than used for our ChIP-seq [23]; [34]. Concerning *cnc*, *cnc_*Col expression reproduced *cnc* expression in the HL, i.e. partly overlapping that of Col (Fig 2B) [26]. Since rapid morphogenetic movements occur in the head between embryonic stages 8 and 12, we compared *cnc_*Col and *cnc_*Col^mut^ expression by ISH. Lower *cnc_Col* ^mut^ expression was observed in the HL, compared to *cnc_*Col, suggesting that Col regulation of *cnc* via *cnc_*Col contributes to robustness of *cnc* expression (Fig 2C and 2D). *Ama*_Col expression also overlaped that of Col in the HL (Fig 2E and 2F). *Ama_*Col^mut^ expression was not detected in the HL, suggesting a direct control of *Ama* activation by Col (Fig 2G and 2H). We then examined endogenous *Ama* transcription and found that it was specifically lost in the HL, in *col* mutant embryos, confirming that this expression is under direct Col control (Fig 2I and 2J). Together, analysis of *Ama*_Col and *cnc_*Col indicated that *Ama* and *cnc* expression is under direct Col regulation. Beyond, it showed that Col binding to embryonic chromatin could identify new genes involved in head development.

### Col targets in neurons

Col is expressed in various subsets of interneurons displaying diverse molecular identities and neurotransmitter phenotypes [31]. *nerfin-1*, a TF essential for the expression of a subset of axon guidance genes in nascent neurons and expressed in most neuroblasts and nascent neurons [60], was found among Col targets. We found that *nerfin-1_*Col overlaps with the neuronal *nerfin-1*-6 enhancer [61] and is widely active in the VNC at stages 14–15, raising the question of a modular regulation in Col expressing-neurons. We noticed, however, that, beyond stage 15, *nerfin-1_*Col^mut^ expression was lost in a few lateral neurons (S4 Fig), which we identified as EL neurons by Eve antibody staining (data not shown). As mentionned above, Col controls *eve* expression in EL neurons [31], likely directly (S1 Fig). Whether *nerfin-1* regulation by Col is only direct or also contributed by Eve in a feed-forward process is an open question. Several Col peaks were found to overlap with DNA fragments driving reporters active in EL neurons in stage 16 embryos (http://www.janelia.org/gal4-gen1) [62]. It will be interesting to see in the future which of the associated genes are direcly regulated by Col in these neurons.

#### Col directly regulates *ap* and *eya* in peptidergic Ap-neurons

Studies of the transcriptional regulatory network controlling the peptidergic identity of Ap-expressing neurons showed that Col regulates both Ap and Eya expression in the segmental dorsal AP (dAP) neurons and the Tv1-4 neurons which form only in thoracic T1-T3 segments, and *dimmed* (*dimm*, FBgn0023091; a bHLH transcription Factor), expressed in the Tv1 (Tvb) neuron, from stage 16 [28]. Col, Ap, Eya, and Dimm act together to activate Dopamine D1 Receptor (DopR) and Neuropeptide-like precursor 1 (Nplp1) specifically in Tv1 and, possibly the dAp neuron, thereby specifying their peptidergic identity. Although this cascade represents one of the best-studied neuron subtype transcription networks [28]; [52], direct transcriptional regulations remained to be established. We did not detect Col peaks in either *dimm*, *DopR* or *nplp1*, suggesting that regulation by Col was indirect or occurred later than stages used for ChIP-seq. However, the presence of Col peaks in *ap* and *eya* suggested a direct regulation, especially since the *ap* Col peak mapped within apC, an upstream region driving neuronal *ap* expression [63]; [28] (Fig 3A). *ap_*Col reproduced *ap* expression in the dAp and 4 Tv neurons in stage 15 embryos (Fig 3B). Immunostaining for *ap_*Col and either Col or Eya at stage 16 confirmed that only Tv1 and dAp maintain Col expression [28]; [52] (Fig 3D and 3E), confirming transient Col activity in the other Tv neurons. *ap_*Col encompasses two close Col binding sites which were both mutated in *ap_*Col^mut^ (S2 Table). *ap_*Col^mut^ expression was drastically reduced, when detected, in Tv and dAP neurons (Fig 3C, 3F and 3G), indicating that regulation of *ap* by Col in these neurons is direct.

Concerning *eya*, two nearby Col peaks were identified in the first intron, each centered on a Col consensus recognition site (Fig 4A and S2 Table). *eya_*Col was designed to encompass both sites. It reproduced *eya* expression in the dAp and Tv neurons at stage 16, i.e., at a later developmental stage than *ap_*Col (Fig 4B and 4C). When both Col sites were mutated, *eya_*Col^mut^ expression was completely lost in dAp but only reduced in the Tv1-4 cluster (Fig 4D and 4E), indicating that Col is required for *eya* activation in dAp neurons, and up-regulation in Tv neurons. We then mutated separately each predicted Col site. *eya_*Col^mut.2^ showed no expression in dAP neurons while *eya_*Col^mut.1^ was still expressed, although more weakly than *eya_*Col (Fig 4F and 4G). We thus conclude that Col site 2 is strictly required for *eya* transcription in dAP neurons, while Col site 1 could confer robustness to regulation by Col. In conclusion, Col binding to chromatin showed that it directly controls *ap* and *eya* expression in specific subsets of neurons and identified the related CRMs.

### Col targets in dorso-lateral muscles

Col is transiently expressed in muscle PCs at the origin of DL muscles, before being uniquely maintained in the elongating DA3 muscle. Time-series datasets for Mef2 and RNA Pol II binding to chromatin and marks of open chromatin and/or active enhancers, H3K79me3 and H3K27ac [64]; [65]; [66] were suggestive of *eya_*Col, *Mrtf*_Col, *Oaz_*Col and *so_*Col activity in the mesoderm (S2 Table). We found that *eya_*Col was stochastically expressed in the DA3 muscle, mainly in thoracic segments (see below, Fig 5A). *so_*Col and *Oaz_*Col were expressed in the DA3 and other DL muscles. The expression pattern of *so_*Col^mut^ did not, however, significantly differ from that of *so_*Col (S5 Fig) while *Oaz_*Col^mut^ expression was lost (S6 Fig). Lastly, *Mrtf*_Col expression was observed in virtually all muscles, but not altered upon mutation of the Col binding site (S7 Fig). In two cases, *eya_*Col and *Oaz_*Col, their expression was dependent upon the predicted Col binding site. In 2 other cases, *so_*Col and *Mrtf*_Col, a direct regulatory role for Col binding was left uncertain.

### Cross-regulation between *col* and *eya* reveals new complexity in the muscle specification process

*eya_*Col expression was also observed in the DA3 muscle. Furthermore, this expression was lost with *eya_*Col^mut^ (Fig 5A and 5B) and down-regulated by mutating either Col site 1 or 2 (data not shown). *eya* was previously reported to be broadly expressed in the somatic mesoderm at stage 9, and assigned a rather general function in modulating somatic myogenesis [67], while *col* was only required in DL muscles [32]. The *eya* and *col* mutant phenotypes and *eya_*Col expression evoked the possibility of *eya*/*col* cross-regulation in DL muscle lineages. Immunostaining stage 11 embryos for both Col and Nautilus (Nau), to identify muscle PCs and FCs [68], together with *eya* ISH, revealed that *eya* and Col are co-expressed in DL PCs, including the PC giving rise to the DA3 muscle (Fig 5C and 5C’). We therefore examined *eya* expression in *col* ^*1*^ mutants. To circumvent the lack of detectable Col protein in these mutants, we used the early mesodermal *col* CRM (col^ECRM^-LacZ; [69] to follow *col*-expressing myoblasts (Fig 5D and 5D’). Triple staining for β-gal, Nau and *eya* showed that *eya* expression was strongly decreased, when not lost, in *col* mutant DL PCs, showing that Col regulates *eya* transcription at that stage (Fig 5E and 5E’). Since a DA3 phenotype has not been previously noted, we re-examined the *eya* DL muscle phenotype [67]. Loss of LL1, and malformation of DA3 muscles was observed with high frequency in *eya* ^*cli-IID*^ */Df(2L)BSC354* embryos completely lacking *eya* function (Fig 5F–5H). This was correlated with the loss of muscle Col expression (Fig 5I and 5J), which could be traced back to stage 11, during the process of selection of PCs from the Col-expressing promuscular cluster (Fig 5K and 5L). These results indicated that *eya* activity was required for high level *col* expression at that step. Together, the reciprocal changes of *eya* and *col* expression in *col* and *eya* mutants and related muscle defects, led us to conclude that regulation of *eya* by Col in muscle PCs is direct, and that cross-regulation between *col* and *eya* genes is critical for establishing the *Drosophila* muscle pattern.

**Fig 6:**
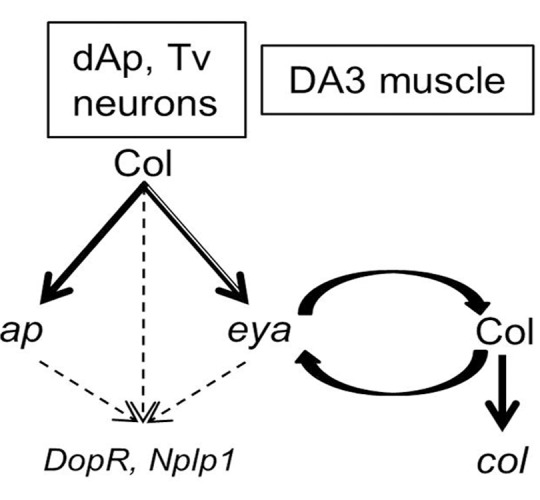
Scheme for Col direct regulation of *ap* and *eya* in specific neuron and muscle lineages. Col directly controls *ap*_Col and *eya*_Col CRM activity in the Tv and dAP neurons and *eya*_Col and *col*_Col CRM in the DA3 muscle lineage. The two Col binding sites in *eya*_Col CRM are not functionally equivalent (double arrow). *eya* and *col* cross-regulate each other in the DA3 PC. Control of *DopR* and *Nplp1* expression in Tv and dAP neurons (Baumgardt et al., 2007) could be indirect.

Col appears to directly regulate *eya* in specific muscle and neuronal lineages, via binding to the same genomic sites (Fig 6). This provides the first example of diversification of Col regulatory functions, through differential regulation of the same gene in different cell contexts.

## Discussion

Col is dynamically expressed and is required in various developmental processes during *Drosophila* embryogenesis. Through genome-wide mapping of Col chromatin binding sites, we identified direct Col targets, among which transcription regulators, indicating that Col contributes both directly, and through activation of other TFs, to various TF combinatorial codes defining cell identities. Examples are the direct regulation of *ap* and *eya* in specific Ap neurons and the cross-regulation brought to light here, between *eya* and *col*, during specification of dorso-lateral muscle identity.

### Col direct gene targets

ChIP-Seq analysis of chromatin from 10–12h old embryos identified 559 Col binding peaks, corresponding to 415 potential Col direct target genes. Studies of 10 targets revealed regulation by Col in different cell-types, confirming the versatility of Col regulatory functions. Of note, our selection of Col targets included genes with known expression profiles and was therefore biased towards positive regulation. It does not exclude that Col could repress some of its targets. 792 regions binding Col/Kn were independently identified in 0 to 12h embryos by the modENCODE cis-regulatory annotation project [70]. The modENCODE genomic regions did not significantly overlap with the positions of Col peak summits mapped here, however, and we could not detect an enrichment of the EBF/Col motif in the modENCODE peak collection. One possibility to explain this lack of overlap is the different source of antibodies used for ChIP. Another genome-wide search for Col/Kn chromatin binding sites was performed in 3^rd^ instar larvae by the DamID method [59]. 99 genes were found to be both bound and regulated by Col in class IV md neurons, with a broad range of molecular functions possibly contributing to dendritic arbor formation [59]. 15 were found among our 415 genes set (S1 Table), suggesting that they are bound by Col already in embryos. Another set of 34 “neuronal” genes bound by Col in larvae, though not regulated by Col in md neurons, is bound by Col in embryos. (S1 Table) [59]. 9 of them code for TFs, including *nerfin-1*, for which we obtained evidence of direct regulation by Col in EL neurons. It will be interesting to determine in which neuron subtypes, TFs bound by Col in embryos and larvae are expressed, as potential entry sites to new networks controlling neuron identity from embryos to larvae. A study in the nematode *C*. *elegans* concluded that the COE factor UNC-3 was a master regulator of « cholinergic genes » in motor neurons [21]. Our ChIP-seq data failed to detect Col binding to either the *Acetylcholine esterase*, or *Acetylcholine receptor* genes, or the *choline acetyl transferase* upstream region driving expression in cholinergic neurons [71], correlating with the observation that Col is expressed in one cholinergic interneuron per hemisegment, but not in embryonic motor neurons. Among the Col-expressing neurons, the best characterized are Tv1 and dAp. CRM activity demonstrated that both *ap* and *eya* expression is under independent, direct Col control in these neurons, with *ap_*Col being active earlier than *eya_*Col. Col requirement for *ap* and *eya* expression in the Tv1 neuron persists to the adult stage [52]. It will be interesting to see whether the same CRMs remain active from embryo to adult.

### Context-dependent Col binding to chromatin and gene regulation

Over-expression experiments showed that Col ability to auto-regulate or activate target genes is highly cell-context dependent, indicating a need to co-operate with other TFs [23]; [33]; [25]. The consensus CCCnnGGGA motif was found close to the center of a large majority of Col ChIP-seq peaks, showing that Col binds *in vivo* preferentially, if not exclusively, to this motif in the chromatin of different cell types. No other significantly enriched motif was found by MEME analysis of the whole set of 559 Col peaks. This can be related to the diversity of expression profiles and therefore cis-regulatory signatures of Col-regulated genes. Mutating the Col binding site resulted into either loss (e.g., *ap*, *Ama*, *eya*) or decrease (e.g., *cnc*) of CRM activity, suggesting that Col either activates or up-regulates/maintains gene transcription, depending upon the gene and cell type/developmental process. In other cases, such as *so* and *Mrtf*, CRM activity failed to reveal a requirement for Col binding, raising the possibility that Col could prime some genes in the chromatin for their later regulation by other lineage-specific transcription factors, as proposed for EBF [44]; [72]. The genome distribution of Col peaks shows cases of several peaks associated with the same gene (S1 Table), for example *eya*. Independent mutation of each Col binding site in *eya_*Col showed that one site is strictly required for CRM activity in dAp neurons, while the other provides robustness to this activity. Both sites contribute to CRM activity in Tv neurons and the DA3 muscle. The presence of several Col peaks can therefore reflect redundance of cis-elements required for robustness/precision of Col regulation in a given cell, or/and differential regulation in different cell types, with possible evolutionary implications.

### New loops in transcriptional regulatory networks controlling skeletal muscle identity

*eya_*Col activity revealed that *eya* is a direct target of Col in DL muscle PCs. Previously, *eya* was assigned a rather general role in modulating somatic myogenesis, downstream of the myogenic factor Tinman (Tin)/Nkx2.5 [67], while *col* was shown to be negatively regulated by Tin and Tail-up/Islet1 in dorsal muscles and cooperate with Nautilus/D-MyoD in DL muscles [68]; [32]. Our discovery of *col/eya* cross regulation in DL muscle PCs therefore brings to light a new layer of intricacy in the transcriptional control of muscle identity, with evolutionary implication. Indeed, cooperation between Eya1/2, Six1/4 and MyoD (Mrf), and Ebfs and MyoD has been shown to operate during vertebrate myogenesis [73]; [74]; [75]; [76]; [18]. Future investigation of epistatic interactions between muscle iTFs such as Col and myogenic regulators such as *eya*, *Six*, and Mrf(s) in patterning the *Drosophila* somatic musculature, should provide deeper insight into which aspects of the myogenic regulatory network have been conserved and/or diversified during evolution. The question of ancestral functions of COE proteins, which appeared with metazoans [7], is highly speculative, in view of COE pleiotropic functions in extant phyla ([8]; [31]; [77], and references in the above text). Col direct regulation of *eya*, both in specific muscle and neuronal lineages, may illustrate one mechanism behind the evolutionary diversification of Col biological roles.

## Supporting Information

S1 Fig The Col peak in *eve*. Annotation of the Col peak in *eve*, adapted from *Gene Browser* (GEO submission GSE67805). 17.5 kb of the *eve* genomic region are shown (Chr2R: 5.859.800–5.877.300) with the Flybase gene annotation indicated by blue bars (transcribed regions) and intervening lines (introns). Black arrows indicate the direction of transcription of *cnc* and cg12134. The Col Dam-ID binding regions [59] are indicated by yellow bars, top line. The summit of the ChIP-Col peak identified by SISSRs and position of the Col binding site(s) identified by MEME are indicated by blue and violet lines, respectively. The position of EveEL is indicated by a blue box. Scale is indicated.
(TIF)

S2 Fig *col* direct autoregulation. (A,B) Col expression in A, stage 11 and B, stage 16 embryos. (C,D) col2.3–0.9 expression. (E,F) col2.3–0.9^mut^ expression. A,C,E: ventral view; B,D,F: lateral view. Mutation of the Col binding site specifically affects col2.3–0.9^mut^ expression in the DA3 muscle (arrow).
(TIF)

S3 Fig Col regulates *tkv_*Col and *jing_*Col expression in the developing lymph gland. (A-H) In situ hybridization to *GFP* transcripts in stage 14 (A,B,E,F) and 16 (C,D,G,H) *tkv_*Col (A,C), *tkv_*Col^mut^ (B,D), *jing_*Col (E,G) and *jing_*Col^mut^ (F,H) embryos. Col regulates both *tkv_*Col (A-D) and *jing_*Col (E-H) activity specifically in the developing LG (white arrow). Dorsal views.
(TIF)

S4 Fig Col regulates *nerfin-1*-_Col expression in EL neurons. (A) Annotation of the Col peak in *nerfin-1*, with the same representation as in Fig 2A; 10.6 kb of the *nerfin-1* genomic region are shown (Chr3L: 903.800–914.400). The position of the previously characterized nerfin-1-6 enhancer is indicated by a blue box. (B, C) Staining of stage 15 *nerfin-1*_Col (B) and *nerfin-1*_Col^mut^ (C) embryos for Col (red), and GFP (green). Only Col and GFP stainings are shown in white in B’,C’ and B”,C”, respectively. A close up view of the squared area (3 segments) is shown below in each panel. The white arrow points to the *nerfin-1*_Col site of expression lost in *nerfin-1*_Col^mut^ embryos.
(TIF)

S5 Fig *so_*Col expression in developing DL muscles. (A-D) Staining for moeGFP expression of stage 14 (A,C) and stage 16 (B,D) *so_*Col (A,B) and *so_*Col^mut^ (C,D) embryos. The brackets in A,C indicate the position of the DL muscles. The DA3, DO5 and LL1 muscles are indicated in some segments in B,D, by an asterisk, a vertical arrow and a double asterisk, respectively. Lateral views.
(TIF)

S6 Fig Col regulates Oaz_Col expression in the DA3 muscle. (A,B) View of one abdominal segment of stage 13 (A) and stage 15 (B) Oaz_Col embryo stained for Col (DA3 muscle, red) and moeGFP (green); only moeGFP staining is shown on the right, in white. Oaz_Col is expressed in the DA3 and other DL muscles. Staining of stage 14 (C,E) and 16 (D,F) Oaz_Col (C,D) and Oaz_Col^mut^ (E,F) embryos. The bracket in C indicates the position of DL muscle precursors expressing Oaz_Col. Oaz_Col^mut^ expression is not detected in DL muscles. The DA3 muscle is indicated by an asterisk in D.
(TIF)

S7 Fig *Mrtf*_Col expression in somatic muscles. (A-D) Staining of stage 17 *Mrtf*_Col (A,B) and *Mrtf*_Col^mut^ (C,D) embryos for Col (red) and moeGFP (green); only moeGFP staining is shown in B,D. The DA3 muscle is indicated in some segments by an asterisk. Lateral views.
(TIF)

S1 File Analyses of Col-ChIP seq data. **ChIP-seq summits and MEME sites.** DATASET: GEO accession number: GSE 67805.
(TXT)

S1 Table 415 genes bound by Col *in vivo*. (A) Gene Name. (B) Number of in vivo Col peaks. (C) Chromosomal position and height of each peak. (D) Peaks genomic coordinates. (E) Annotation symbol. (F) Flybase ID number.
(PDF)

S2 Table CRM_Col constructs and mutated Col binding sites. (PDF)
