# Supplementary figures and images for: Genome-Wide Mapping of Collier In Vivo Binding Sites Highlights Its Hierarchical Position in Different Transcription Regulatory Networks
Source: PLoS One. 2015 Jul 23;10(7):e0133387. doi: 10.1371/journal.pone.0133387 (PMC4512700; doi:10.1371/journal.pone.0133387)

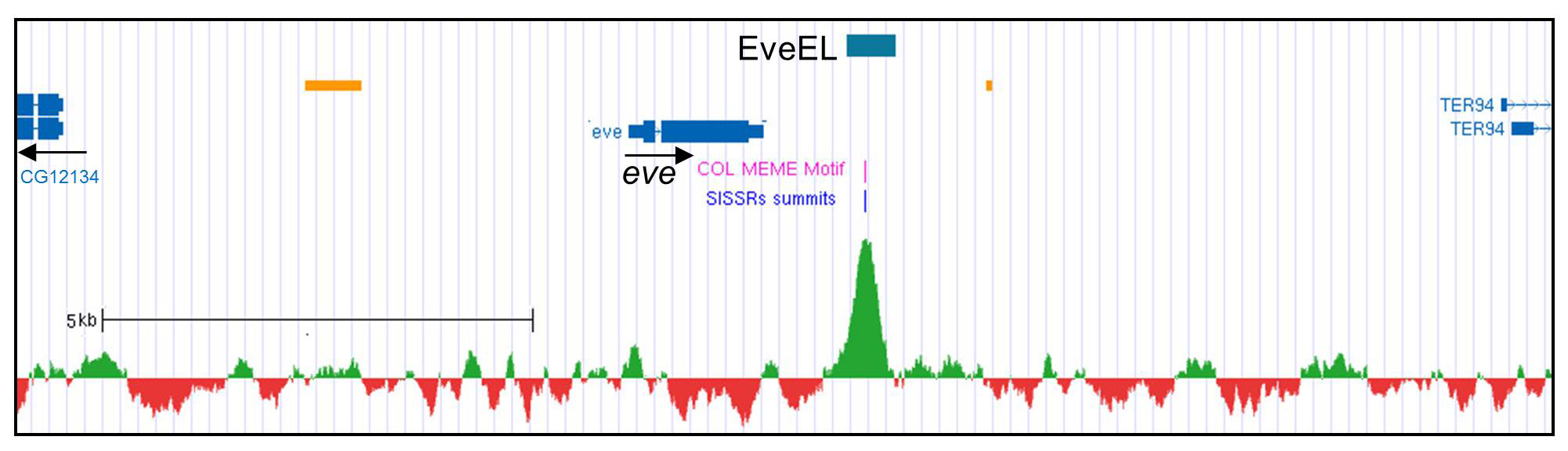

Supplement: S1 Fig — Annotation of the Col peak in eve, adapted from Gene Browser (GEO submission GSE67805). 17.5 kb of the eve genomic region are shown (Chr2R: 5.859.800–5.877.300) with the Flybase gene annotation indicated by blue bars (transcribed regions) and intervening lines (introns). Black arrows indicate the direction of transcription of cnc and cg12134. The Col Dam-ID binding regions [59] are indicated by yellow bars, top line. The summit of the ChIP-Col peak identified by SISSRs and position of the Col binding site(s) identified by MEME are indicated by blue and violet lines, respectively. The position of EveEL is indicated by a blue box. Scale is indicated. (TIF) [file pone.0133387.s001.tif]

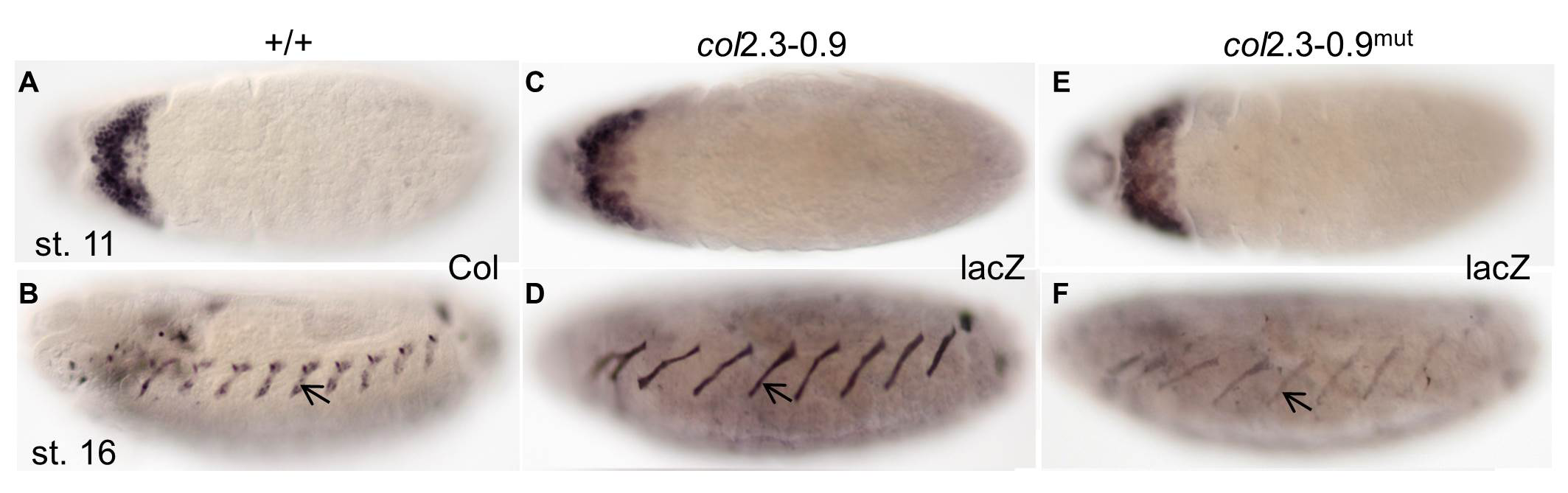

Supplement: S2 Fig — (A,B) Col expression in A, stage 11 and B, stage 16 embryos. (C,D) col2.3–0.9 expression. (E,F) col2.3–0.9mut expression. A,C,E: ventral view; B,D,F: lateral view. Mutation of the Col binding site specifically affects col2.3–0.9mut expression in the DA3 muscle (arrow). (TIF) [file pone.0133387.s002.tif]

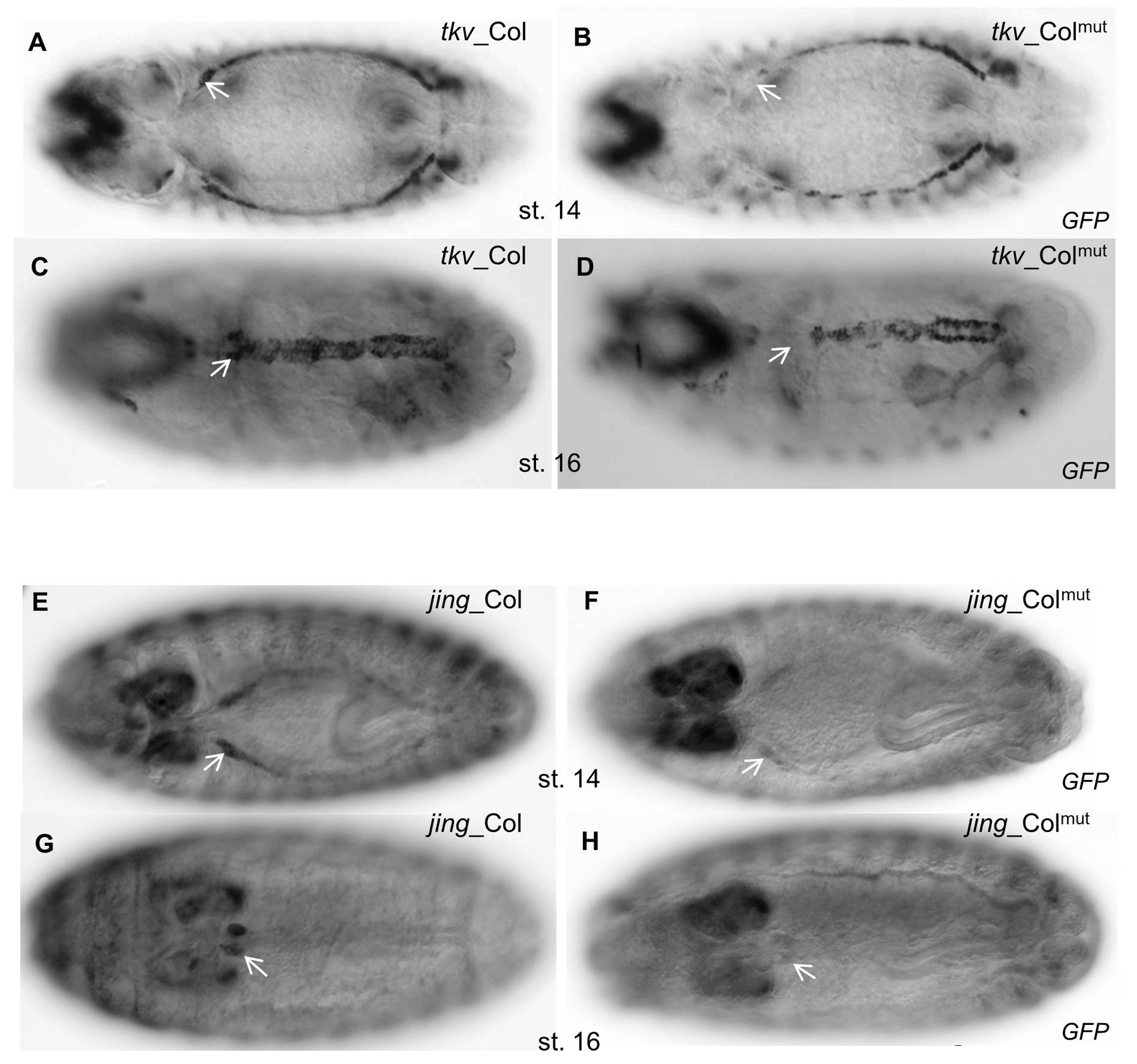

Supplement: S3 Fig — (A-H) In situ hybridization to GFP transcripts in stage 14 (A,B,E,F) and 16 (C,D,G,H) tkv_Col (A,C), tkv_Colmut (B,D), jing_Col (E,G) and jing_Colmut (F,H) embryos. Col regulates both tkv_Col (A-D) and jing_Col (E-H) activity specifically in the developing LG (white arrow). Dorsal views. (TIF) [file pone.0133387.s003.tif]

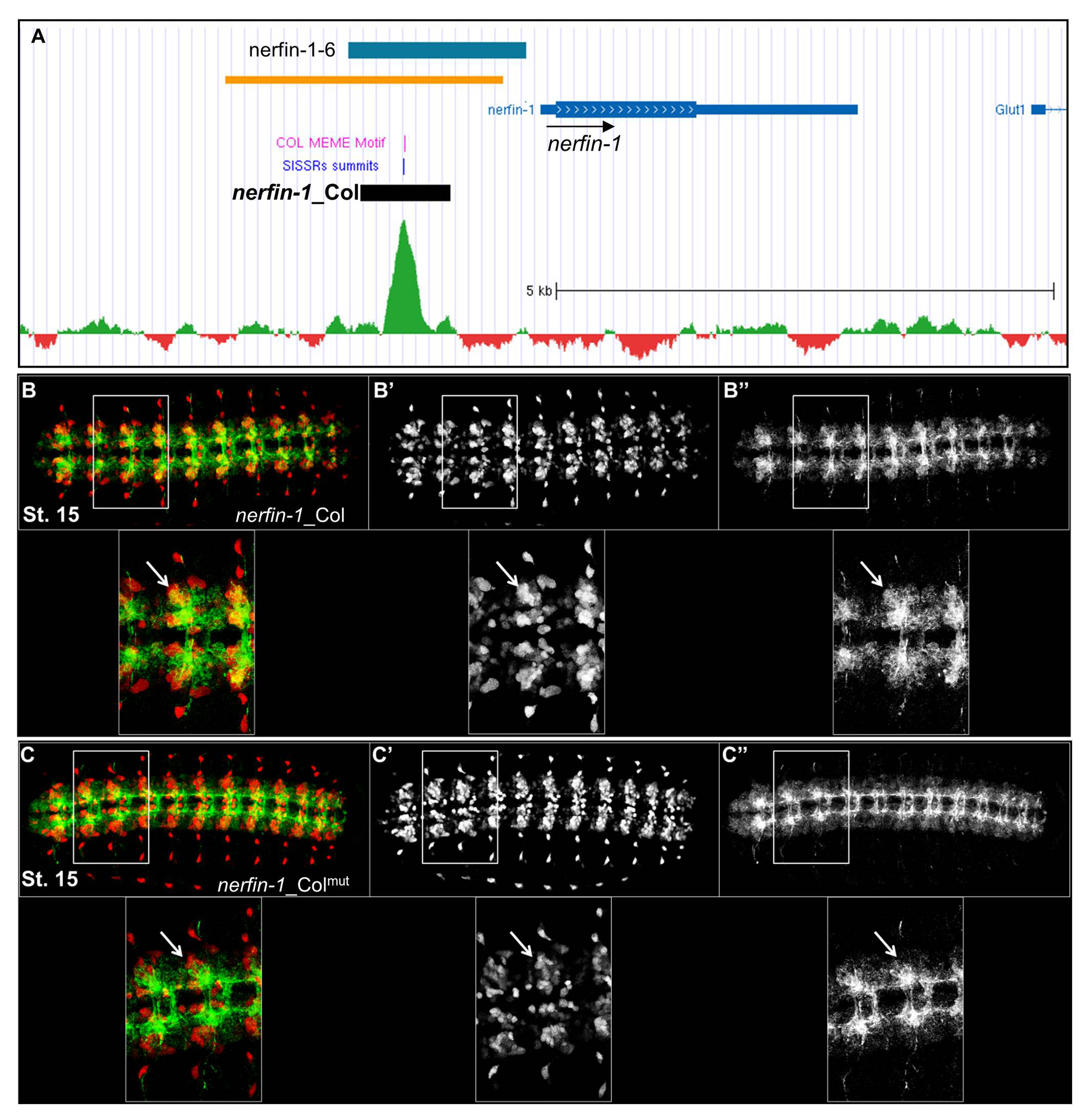

Supplement: S4 Fig — (A) Annotation of the Col peak in nerfin-1, with the same representation as in Fig 2A; 10.6 kb of the nerfin-1 genomic region are shown (Chr3L: 903.800–914.400). The position of the previously characterized nerfin-1-6 enhancer is indicated by a blue box. (B, C) Staining of stage 15 nerfin-1_Col (B) and nerfin-1_Colmut (C) embryos for Col (red), and GFP (green). Only Col and GFP stainings are shown in white in B’,C’ and B”,C”, respectively. A close up view of the squared area (3 segments) is shown below in each panel. The white arrow points to the nerfin-1_Col site of expression lost in nerfin-1_Colmut embryos. (TIF) [file pone.0133387.s004.tif]

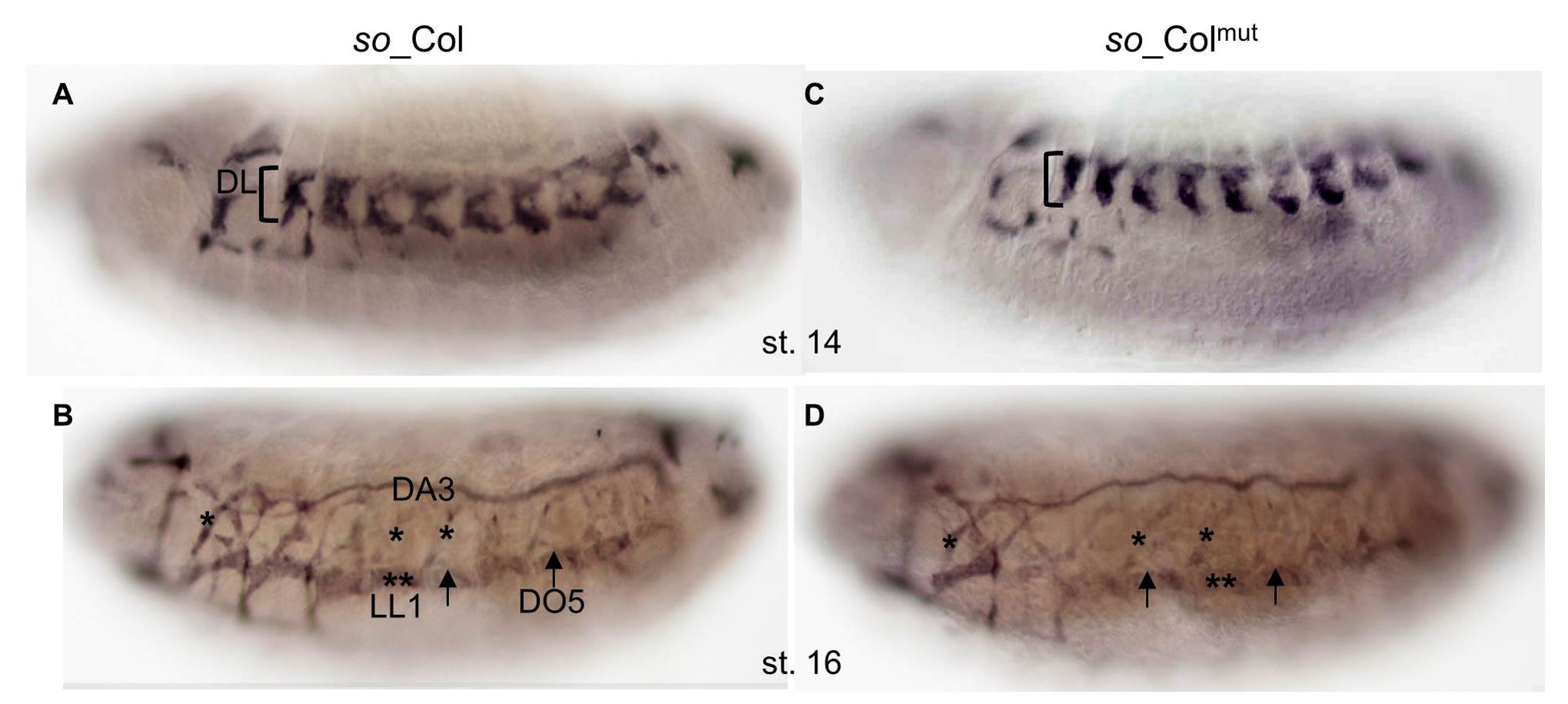

Supplement: S5 Fig — (A-D) Staining for moeGFP expression of stage 14 (A,C) and stage 16 (B,D) so_Col (A,B) and so_Colmut (C,D) embryos. The brackets in A,C indicate the position of the DL muscles. The DA3, DO5 and LL1 muscles are indicated in some segments in B,D, by an asterisk, a vertical arrow and a double asterisk, respectively. Lateral views. (TIF) [file pone.0133387.s005.tif]

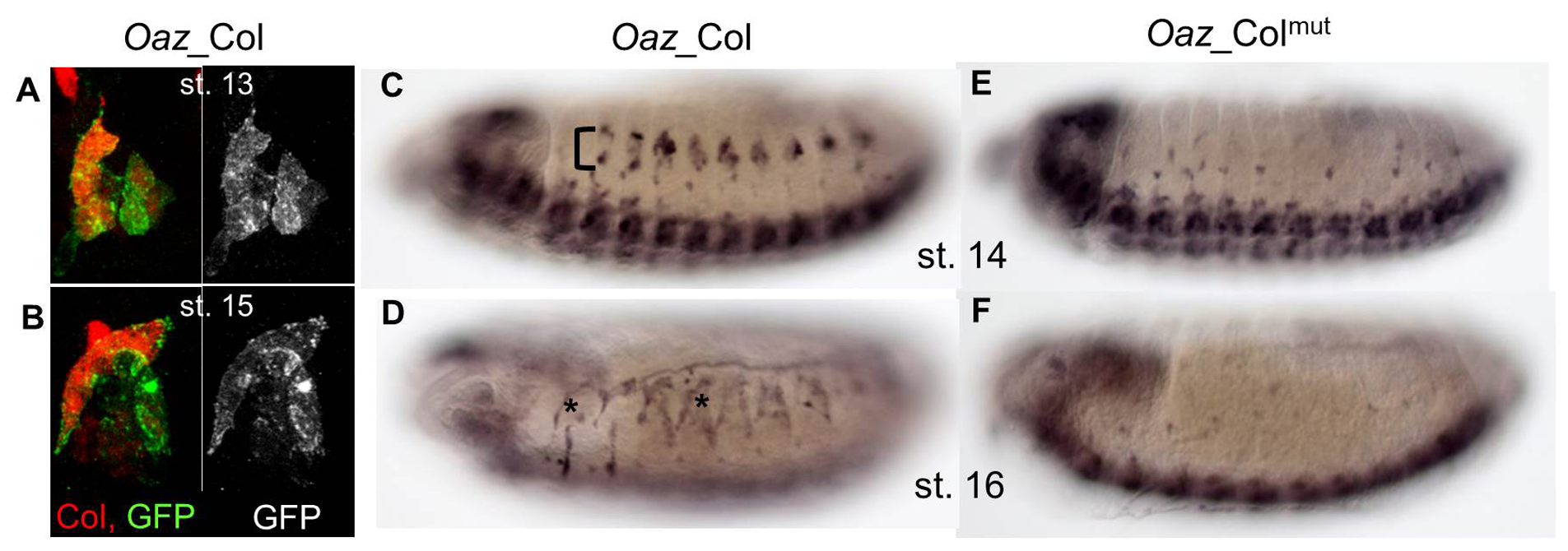

Supplement: S6 Fig — (A,B) View of one abdominal segment of stage 13 (A) and stage 15 (B) Oaz_Col embryo stained for Col (DA3 muscle, red) and moeGFP (green); only moeGFP staining is shown on the right, in white. Oaz_Col is expressed in the DA3 and other DL muscles. Staining of stage 14 (C,E) and 16 (D,F) Oaz_Col (C,D) and Oaz_Colmut (E,F) embryos. The bracket in C indicates the position of DL muscle precursors expressing Oaz_Col. Oaz_Colmut expression is not detected in DL muscles. The DA3 muscle is indicated by an asterisk in D. (TIF) [file pone.0133387.s006.tif]

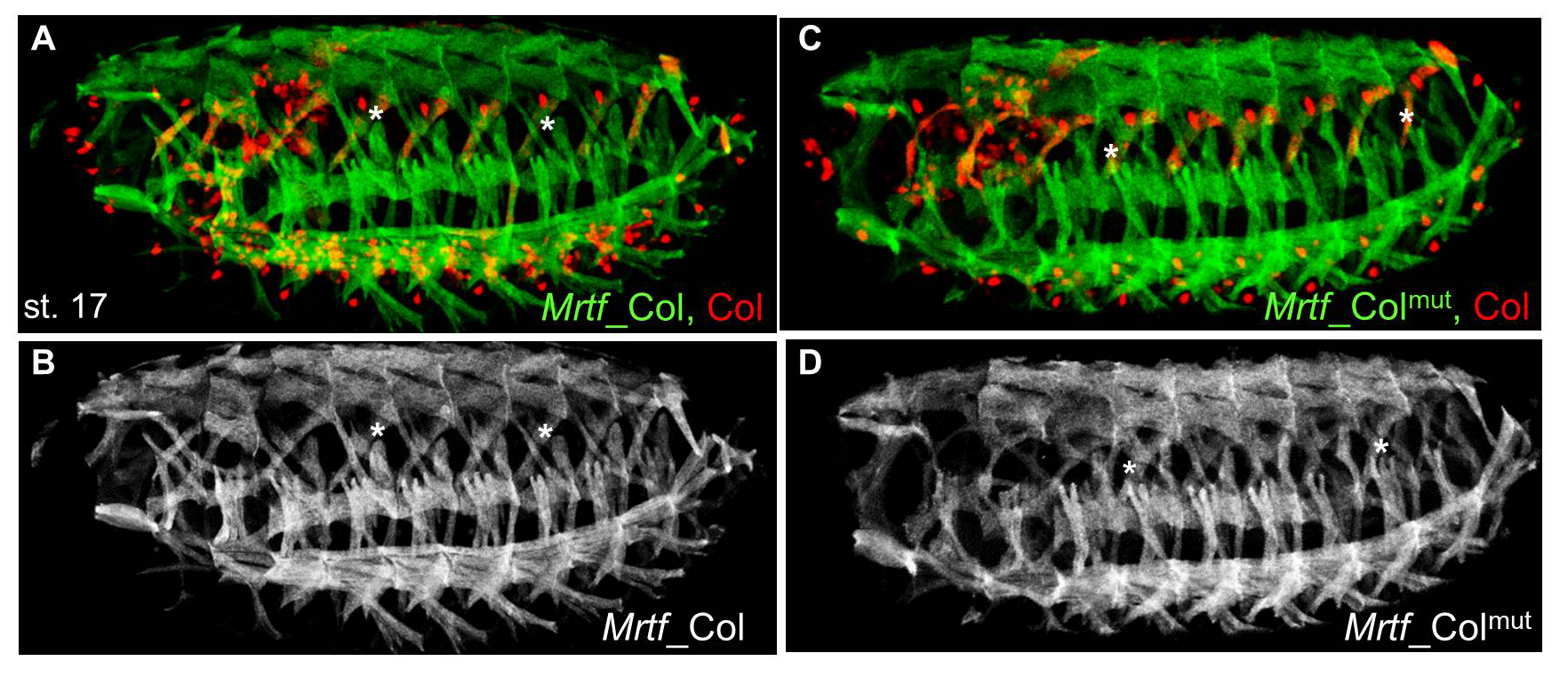

Supplement: S7 Fig — (A-D) Staining of stage 17 Mrtf_Col (A,B) and Mrtf_Colmut (C,D) embryos for Col (red) and moeGFP (green); only moeGFP staining is shown in B,D. The DA3 muscle is indicated in some segments by an asterisk. Lateral views. (TIF) [file pone.0133387.s007.tif]
