# Supplementary material for: Genome-Wide Mapping of Collier In Vivo Binding Sites Highlights Its Hierarchical Position in Different Transcription Regulatory Networks
Source: PLoS One. 2015 Jul 23;10(7):e0133387. doi: 10.1371/journal.pone.0133387 (PMC4512700; doi:10.1371/journal.pone.0133387)
Supplement: S1 Table — (A) Gene Name. (B) Number of in vivo Col peaks. (C) Chromosomal position and height of each peak. (D) Peaks genomic coordinates. (E) Annotation symbol. (F) Flybase ID number. (PDF) [file pone.0133387.s009.pdf]

Table S1: 415 genes bound by Col in vivo.

/ peak overlapping two genes
#intragenic for nub/5'side for pdm2
*Curated annotation from Kvon et al.,Nature,2014 (Supplementary table 4)
**Curated annotation from REDfly 3.3

Gene name	Number of Col peaks	Peaks positions and heights	Peaks cooordinates	Gene Annotation symbol	Flybase ID
sick	9	sick_Intragenic_(4,1)<br>sick_Intragenic_(4)<br>sick_Intragenic_(3,68)<br>sick_Intragenic_(3,31)<br>sick_Intragenic_(3,13)<br>sick_Intragenic_(2,53)<br>sick_Intragenic_(2,41)<br>sick_Intragenic_(2,29)<br>sick_Intragenic_(2,19)	chr2L:19829551-19829552<br>chr2L:19829831-19829832<br>chr2L:19908161-19908162<br>chr2L:19802561-19802562<br>chr2L:19893371-19893372<br>chr2L:19837151-19837152<br>chr2L:19831581-19831582<br>chr2L:19838841-19838842<br>chr2L:19807211-19807212	CG43720	FBgn0263873
sli	7	sli_Intragenic_(7,51)<br>sli_Intragenic_(4,68)<br>sli_Intragenic_(4)<br>sli_Intragenic_(2,87)<br>sli_Intragenic_(2,62)<br>sli_Intragenic_(2,52)<br>sli_Intragenic_(2,2)	chr2R:11783131-11783132<br>chr2R:11800571-11800572<br>chr2R:11798141-11798142<br>chr2R:11779551-11779552<br>chr2R:11808281-11808282<br>chr2R:11800021-11800022<br>chr2R:11799411-11799412	CG43758	FBgn0264089
Oaz	6	Oaz_Intragenic_(16,17)<br>Oaz_5' side_(8,51)<br>Oaz_Intragenic_(5,12)<br>Oaz_Intragenic_(2,23)<br>Oaz_Intragenic_(2,15)<br>Oaz_Intragenic_(2,02)	chr2R:10325311-10325312<br>chr2R:10323251-10323252<br>chr2R:10340171-10340172<br>chr2R:10327301-10327302<br>chr2R:10327671-10327672<br>chr2R:10333611-10333612	CG42702	FBgn0261613
hth	5	hth_Intragenic_(3,57)<br>hth_Intragenic_(2,96)<br>hth_Intragenic_(2,58)<br>hth_3' side_(2,56)<br>hth_Intragenic_(2,42)	chr3R:6402141-6402142<br>chr3R:6339011-6339012<br>chr3R:6402641-6402642<br>chr3R:6324751-6324752<br>chr3R:6462431-6462432	CG17117	FBgn0001235
CG34380	5	CG34380_Intragenic_(3,11)<br>CG34380_Intragenic_(2,71)<br>CG34380_Intragenic_(2,66)<br>CG34380_Intragenic_(2,26)<br>CG34380_Intragenic_(1,95)	chr2L:6183651-6183652<br>chr2L:6202631-6202632<br>chr2L:6203041-6203042<br>chr2L:6199411-6199412<br>chr2L:6205381-6205382	CG34380	FBgn0085409
olf413	5	olf413_Intragenic_(3,95)<br>olf413_Intragenic_(3,43)<br>olf413_Intragenic_(2,85)<br>olf413_Intragenic_(2,5)<br>olf413_Intragenic_(2,34)	chr3L:22192391-22192392<br>chr3L:22188491-22188492<br>chr3L:22193631-22193632<br>chr3L:22190781-22190782<br>chr3L:22167701-22167702	CG12673	FBgn0037153
snky	4	snky_3' side_(3,68)<br>snky_3' side_(2,83)<br>snky_3' side_(2,81)<br>snky_3' side_(2,09)	chr3L:13182171-13182172<br>chr3L:13182711-13182712<br>chr3L:13187131-13187132<br>chr3L:13181881-13181882	CG11281	FBgn0086916
CG44014	4	CG44014_5' side_(4,8)<br>CG44014_5' side_(3,13)<br>CG44014_5' side_(2,87)<br>CG44014_5' side_(2,78)	chr3R:11338151-11338152<br>chr3R:11344411-11344412<br>chr3R:11338391-11338392<br>chr3R:11344051-11344052	CG44014	FBgn0264776
Antp	4	Antp_Intragenic_(5,22)<br>Antp_5' side_(4,72)<br>Antp_Intragenic_(2,87)<br>Antp_Intragenic_(2,37)	chr3R:2746971-2746972<br>chr3R:2834151-2834152<br>chr3R:2778591-2778592<br>chr3R:2816851-2816852	CG1028	FBgn0260642
px	4	px_Intragenic_(9,48)<br>px_Intragenic_(3,46)<br>px_Intragenic_(2,32)<br>px_Intragenic_(2,24)	chr2R:18420911-18420912<br>chr2R:18399351-18399352<br>chr2R:18412951-18412952<br>chr2R:18398041-18398042	CG4444	FBgn0003175
jing	4	jing_Intragenic_(6,06)<br>jing_Intragenic_(5,2)<br>jing_Intragenic_(5,17)<br>jing_Intragenic_(4,94)	chr2R:2426211-2426212<br>chr2R:2421711-2421712<br>chr2R:2482581-2482582<br>chr2R:2447621-2447622	CG9397	FBgn0086655
CG42342	4	CG42342_Intragenic_(4,38)<br>CG42342_Intragenic_(4,37)<br>CG42342_Intragenic_(3,23)<br>CG42342_Intragenic_(2,01)	chr3R:12391431-12391432<br>chr3R:12330171-12330172<br>chr3R:12386031-12386032<br>chr3R:12378161-12378162	CG42342	FBgn0259244
pum	4	pum_Intragenic_(7,83)<br>pum_Intragenic_(5,74)<br>pum_Intragenic_(3,86)<br>pum_Intragenic_(3,43)	chr3R:4970751-4970752<br>chr3R:4909131-4909132<br>chr3R:5007181-5007182<br>chr3R:4953951-4953952	CG9755	FBgn0003165
drl	3	drl_Intragenic_(2,95)<br>drl_3' side_(2,6)*<br>drl_Intragenic_(2,11)	chr2L:19202581-19202582<br>chr2L:19236521-19236522<br>chr2L:19199741-19199742	CG17348	FBgn0015380
CG42238	3	CG42238_Intragenic_(3,07)<br>CG42238_3' side_(2,98)<br>CG42238_Intragenic_(2,8)	chr2L:21001451-21001452<br>chr2L:20951751-20951752<br>chr2L:20998911-20998912	CG42238	FBgn0250867
CG43202	3	CG43202_3' side_(3,6)<br>CG43202_5' side_(2,37)<br>CG43202_3' side_(2,05)	chr2R:14268471-14268472<br>chr2R:14262961-14262962<br>chr2R:14266241-14266242	CG43202	FBgn0262838
CG42831	3	CG42831_3' side_(6,96)<br>CG42831_5' side_(2,95)<br>CG42831_5' side_(2,83)	chr3L:10819391-10819392<br>chr3L:10804351-10804352<br>chr3L:10803431-10803432	CG42831	FBgn0262020
CG13898	3	CG13898_5' side_(3,5)<br>CG13898_3' side_(3,11)<br>CG13898_3' side_(2,56)	chr3L:815251-815252<br>chr3L:802601-802602<br>chr3L:795511-795512	CG13898	FBgn0035161
CG1789	3	CG1789_3' side_(6,44)<br>CG1789_3' side_(5,02)<br>CG1789_3' side_(3,68)	chrX:8621151-8621152<br>chrX:8630751-8630752<br>chrX:8619641-8619642	CG1789	FBgn0030063
CG15283	3	CG15283_5' side_(4,26)<br>CG15283_5' side_(2,95)<br>CG15283_5' side_(2,31)	chr2L:14456611-14456612<br>chr2L:14454631-14454632<br>chr2L:14459681-14459682	CG15283	FBgn0028844
CG15711	3	CG15711_5' side_(3,18)<br>CG15711_5' side_(3,18)<br>CG15711_5' side_(2,2)	chr2R:12258771-12258772<br>chr2R:12259731-12259732<br>chr2R:12256951-12256952	CG15711	FBgn0034122
Best4	3	Best4_5' side_(3,66)<br>Best4_5' side_(3,11)<br>Best4_5' side_(3)	chr3L:15278851-15278852<br>chr3L:15265611-15265612<br>chr3L:15268391-15268392	CG7259	FBgn0036491
CG2022	3	CG2022_5' side_(7,12)<br>CG2022_5' side_(6,51)<br>CG2022_Intragenic_(2,94)	chr3R:862051-862052<br>chr3R:851351-851352<br>chr3R:815711-815712	CG2022	FBgn0037292
OdsH	3	OdsH_Intragenic_(5,7)<br>OdsH_5' side_(4,45)<br>OdsH_5' side_(2,51)	chrX:17693151-17693152<br>chrX:17677721-17677722<br>chrX:17675931-17675932	CG6352	FBgn0026058
Trim9	3	Trim9_Intragenic_(4,82)<br>Trim9_Intragenic_(4,18)<br>Trim9_Intragenic_(2,44)	chr2L:10621121-10621122<br>chr2L:10550561-10550562<br>chr2L:10566711-10566712	CG31721	FBgn0051721

kn	3	kn_Intragenic_(3,55)<br>kn_Intragenic_(3,26)<br>kn_Intragenic_(2,66)	chr2R:10687091-10687092<br>chr2R:10688181-10688182<br>chr2R:10690401-10690402	CG10197	FBgn0001319
unc-5	3	unc-5_Intragenic_(5,09)<br>unc-5_Intragenic_(4,53)<br>unc-5_Intragenic_(3,13)	chr2R:11250491-11250492<br>chr2R:11249331-11249332<br>chr2R:11240911-11240912	CG8166	FBgn0034013
sbb	3	sbb_Intragenic_(3,63)<br>sbb_Intragenic_(3,33)<br>sbb_Intragenic_(2,2)	chr2R:14226631-14226632<br>chr2R:14228931-14228932<br>chr2R:14225321-14225322	CG5580	FBgn0010575
Ten-a	3	Ten-a_Intragenic_(5,99)<br>Ten-a_Intragenic_(5)<br>Ten-a_Intragenic_(2,95)	chrX:12161541-12161542<br>chrX:12113431-12113432<br>chrX:12106991-12106992	CG42338	FBgn0267001
CG17349	2	CG17349_3' side_(7,07)<br>CG17349_3' side_(2,57)	chr2L:19374411-19374412<br>chr2L:19375251-19375252	CG17349	FBgn0032771
CG10195	2	CG10195_3' side_(2,64)<br>CG10195_3' side_(2,6)	chr2L:19481471-19481472<br>chr2L:19480761-19480762	CG10195	FBgn0032787
CG11629	2	CG11629_3' side_(2,4)<br>CG11629_3' side_(2,27)	chr2L:21896451-21896452<br>chr2L:21893151-21893152	CG11629	FBgn0032965
RYBP	2	RYBP_3' side_(6,46)<br>RYBP_3' side_(3,3)	chr2R:18563271-18563272<br>chr2R:18565251-18565252	CG12190	FBgn0034763
CG13323	2	CG13323_3' side_(4,3)<br>CG13323_3' side_(3,76)	chr2R:8917751-8917752<br>chr2R:8915671-8915672	CG13323	FBgn0033788
CG7255	2	CG7255_3' side_(6,51)<br>CG7255_3' side_(2,47)	chr3L:15304491-15304492<br>chr3L:15304961-15304962	CG7255	FBgn0036493
Toll-6	2	Toll-6_3' side_(7)<br>Toll-6_5' side_(2,94)	chr3L:15342011-15342012<br>chr3L:15327091-15327092	CG7250	FBgn0036494
CG33259	2	CG33259_5' side_(2,34)<br>CG33259_3' side_(2,22)	chr3L:15387511-15387512<br>chr3L:15413771-15413772	CG33259	FBgn0036495
msopa	2	msopa_3' side_(3,59)<br>msopa_5' side_(3,03)	chr3L:22077131-22077132<br>chr3L:22075481-22075482	CG14560	FBgn0004414
Trxr-2	2	Trxr-2_3' side_(2,37)<br>Trxr-2_3' side_(2,01)	chr3L:22568361-22568362<br>chr3L:22561671-22561672	CG11401	FBgn0037170
Rh50	2	Rh50_3' side_(4,25)<br>Rh50_3' side_(3,04)	chr3L:4922511-4922512<br>chr3L:4920891-4920892	CG7499	FBgn0028699
abd-A	2	abd-A_3' side_(2,96)<br>abd-A_Intragenic_(2,25)*	chr3R:12629961-12629962<br>chr3R:12637631-12637632	CG10325	FBgn0000014
CG7956	2	CG7956_3' side_(4,74)<br>CG7956_5' side_(2,26)	chr3R:17359891-17359892<br>chr3R:17344691-17344692	CG7956	FBgn0038890
CG43447	2	CG43447_3' side_(2,58)<br>CG43447_3' side_(2,22)	chr3R:23266651-23266652<br>chr3R:23270111-23270112	CG43447	FBgn0263401
Cyp12e1	2	Cyp12e1_3' side_(2,92)<br>Cyp12e1_3' side_(2,15)	chr3R:6321711-6321712<br>chr3R:6319871-6319872	CG14680	FBgn0037817
CG4161	2	CG4161_5' side_(5,37)<br>CG4161_5' side_(3,45)	chr2L:15429871-15429872<br>chr2L:15430251-15430252	CG4161	FBgn0028892
Oli	2	Oli_5' side_(5,08)<br>Oli_5' side_(4,64)	chr2L:17586271-17586272<br>chr2L:17583851-17583852	CG5545	FBgn0032651
tup	2	tup_5' side_(3,77)<br>tup_Intragenic_(2,54)	chr2L:18897331-18897332<br>chr2L:18867991-18867992	CG10619	FBgn0003896
CG17350	2	CG17350_5' side_(6,24)<br>CG17350_5' side_(2,53)	chr2L:19378291-19378292<br>chr2L:19376771-19376772	CG17350	FBgn0032772
ed	2	ed_5' side_(3,39)<br>ed_Intragenic_(2,71)	chr2L:4023581-4023582<br>chr2L:4034081-4034082	CG12676	FBgn0000547
Rca1	2	Rca1_5' side_(4,61)<br>Rca1_5' side_(2,98)	chr2L:6842591-6842592<br>chr2L:6841771-6841772	CG10800	FBgn0017551
CG33467	2	CG33467_5' side_(3,19)<br>CG33467_5' side_(2,65)	chr2R:10959811-10959812<br>chr2R:10960431-10960432	CG33467	FBgn0053467
CG15925	2	CG15925_5' side_(3,58)<br>CG15925_5' side_(2,55)	chr2R:12333021-12333022<br>chr2R:12332571-12332572	CG15925	FBgn0034129
ap	2	ap_5' side_(5,4)<br>ap_5' side_(2,02)*,**	chr2R:1624602-1624603<br>chr2R:1629081-1629082	CG8376	FBgn0267978
mirr	2	mirr_5' side_(2,64)<br>mirr_5' side_(2,3)	chr3L:12686351-12686352<br>chr3L:12681671-12681672	CG10601	FBgn0014343
fz	2	fz_Intragenic_(5,41)<br>fz_5' side_(3,38)	chr3L:14297011-14297012<br>chr3L:14256271-14256272	CG17697	FBgn0001085
CG7304	2	CG7304_5' side_(4,83)<br>CG7304_5' side_(2,02)	chr3L:15671781-15671782<br>chr3L:15666761-15666762	CG7304	FBgn0036527
Mip	2	Mip_Intragenic_(3,09)<br>Mip_5' side_(2,63)	chr3L:17339541-17339542<br>chr3L:17343171-17343172	CG6456	FBgn0036713
CG43335	2	CG43335_5' side_(6,15)<br>CG43335_5' side_(4,09)	chr3R:11442091-11442092<br>chr3R:11443711-11443712	CG43335	FBgn0263040
pxb	2	pxb_5' side_(2,81)<br>pxb_Intragenic_(2,58)	chr3R:11478791-11478792<br>chr3R:11509091-11509092	CG33207	FBgn0053207
CG4704	2	CG4704_5' side_(3,68)<br>CG4704_5' side_(2,48)	chr3R:18687811-18687812<br>chr3R:18671611-18671612	CG4704	FBgn0039029
klg	2	klg_5' side_(3,51)<br>klg_Intragenic_(3,02)	chr3R:18699531-18699532<br>chr3R:18757841-18757842	CG6669	FBgn0017590
laf	2	laf_5' side_(2,48)<br>laf_5' side_(2,27)	chr3R:708391-708392<br>chr3R:712371-712372	CG14660	FBgn0020280
ci	2	ci_5' side_(6,33)<br>ci_5' side_(5,7)	chr4:81301-81302<br>chr4:81371-81372	CG2125	FBgn0004859
Sox102F	2	Sox102F_Intragenic_(3)<br>Sox102F_5' side_(2,41)	chr4:835331-835332<br>chr4:854811-854812	CG11153	FBgn0039938
aret	2	aret_Intragenic_(2,92)<br>aret_Intragenic_(2,36)	chr2L:12252391-12252392<br>chr2L:12263811-12263812	CG31762	FBgn0000114
CadN	2	CadN_Intragenic_(2,59)<br>CadN_Intragenic_(2,51)	chr2L:17764371-17764372<br>chr2L:17723321-17723322	CG7100	FBgn0015609
Lim3	2	Lim3_Intragenic_(3,66)<br>Lim3_Intragenic_(2,66)	chr2L:19103911-19103912<br>chr2L:19090791-19090792	CG10699	FBgn0002023
VGlut	2	VGlut_Intragenic_(6,02)<br>VGlut_Intragenic_(3,71)	chr2L:2404501-2404502<br>chr2L:2393891-2393892	CG9887	FBgn0031424
DIP-theta	2	DIP-theta_Intragenic_(2,44)<br>DIP-theta_Intragenic_(1,97)	chr2L:5628811-5628812<br>chr2L:5631931-5631932	CG31646	FBgn0051646
eya	2	eya_Intragenic_(3,99)<br>eya_Intragenic_(2,39)	chr2L:6541031-6541032<br>chr2L:6540661-6540662	CG9554	FBgn0000320
Rapgap1	2	Rapgap1_Intragenic_(2,64)<br>Rapgap1_Intragenic_(2,17)	chr2L:7560721-7560722<br>chr2L:7539011-7539012	CG44086	FBgn0264895
Snoo	2	Snoo_Intragenic_(3,57)<br>Snoo_Intragenic_(2,42)	chr2L:7963381-7963382<br>chr2L:7933371-7933372	CG34421	FBgn0085450
Sema-2b	2	Sema-2b_Intragenic_(2,74)<br>Sema-2b_Intragenic_(2,41)	chr2R:12296671-12296672<br>chr2R:12293631-12293632	CG33960	FBgn0264273
mbl	2	mbl_Intragenic_(4,13)<br>mbl_Intragenic_(2,61)	chr2R:13223611-13223612<br>chr2R:13206431-13206432	CG33197	FBgn0265487
otp	2	otp_Intragenic_(4,91)<br>otp_Intragenic_(2,02)	chr2R:16779011-16779012<br>chr2R:16777601-16777602	CG10036	FBgn0015524
CG34371	2	CG34371_Intragenic_(4,42)<br>CG34371_Intragenic_(2,67)	chr2R:19090691-19090692<br>chr2R:19085441-19085442	CG34371	FBgn0085400
pk	2	pk_Intragenic_(3,44)<br>pk_Intragenic_(3,33)	chr2R:3046271-3046272<br>chr2R:3058031-3058032	CG11084	FBgn0003090
luna	2	luna_Intragenic_(2,78)<br>luna_Intragenic_(2,55)	chr2R:6943151-6943152<br>chr2R:6981391-6981392	CG33473	FBgn0040765
fas	2	fas_Intragenic_(3,43)<br>fas_Intragenic_(3,41)	chr2R:9609291-9609292<br>chr2R:9625651-9625652	CG17716	FBgn0000633
bru-3	2	bru-3_Intragenic_(2,85)<br>bru-3_Intragenic_(1,97)	chr3L:13644431-13644432<br>chr3L:13759491-13759492	CG43744	FBgn0264001
comm3	2	comm3_Intragenic_(3,09)<br>comm3_Intragenic_(2,65)	chr3L:15607591-15607592<br>chr3L:15610261-15610262	CG42334	FBgn0259236
Mipp1	2	Mipp1_Intragenic_(3,01)<br>Mipp1_Intragenic_(2,19)	chr3L:16564201-16564202<br>chr3L:16562591-16562592	CG4123	FBgn0026061
Ten-m	2	Ten-m_Intragenic_(3,83)<br>Ten-m_Intragenic_(2,17)	chr3L:22321211-22321212<br>chr3L:22355731-22355732	CG5723	FBgn0004449
CG14459	2	CG14459_Intragenic_(2,74)<br>CG14459_Intragenic_(2,34)	chr3L:22596511-22596512<br>chr3L:22595021-22595022	CG14459	FBgn0037171
Ets65A	2	Ets65A_Intragenic_(3,53)<br>Ets65A_Intragenic_(1,99)	chr3L:6097781-6097782<br>chr3L:6099191-6099192	CG7018	FBgn0005658
CG13894	2	CG13894_Intragenic_(2,31)<br>CG13894_Intragenic_(2,25)	chr3L:698011-698012<br>chr3L:697191-697192	CG13894	FBgn0035157

Hs6st	2	Hs6st_Intragenic_(2,44)<br>Hs6st_Intragenic_(2,06)	chr3R:15831211-15831212<br>chr3R:15784281-15784282	CG4451	FBgn0038755
SKIP	2	SKIP_Intragenic_(4,55)<br>SKIP_Intragenic_(2,41)	chr3R:18036091-18036092<br>chr3R:18105381-18105382	CG31163	FBgn0051163
CG34355	2	CG34355_Intragenic_(3,14)<br>CG34355_Intragenic_(2,18)	chr3R:19655271-19655272<br>chr3R:19660851-19660852	CG34355	FBgn0085384
CG11873	2	CG11873_Intragenic_(2,44)<br>CG11873_Intragenic_(2,05)	chr3R:24894021-24894022<br>chr3R:24910181-24910182	CG11873	FBgn0039633
sima	2	sima_Intragenic_(6,51)<br>sima_Intragenic_(2,37)	chr3R:25936541-25936542<br>chr3R:25916121-25916122	CG45051	FBgn0266411
Gprk2	2	Gprk2_Intragenic_(2,62)<br>Gprk2_Intragenic_(1,95)	chr3R:27276151-27276152<br>chr3R:27233351-27233352	CG17998	FBgn0261988
Dtg	2	Dtg_Intragenic_(5,66)<br>Dtg_Intragenic_(2,03)	chr3R:8475231-8475232<br>chr3R:8475591-8475592	CG6234	FBgn0038071
X11Lbeta	2	X11Lbeta_Intragenic_(4,5)<br>X11Lbeta_Intragenic_(3,16)	chrX:10577271-10577272<br>chrX:10588901-10588902	CG32677	FBgn0052677
Vsx2	2	Vsx2_Intragenic_(5,74)<br>Vsx2_Intragenic_(4,58)	chrX:5435771-5435772<br>chrX:5432761-5432762	CG33980	FBgn0263512
CG13137	1	CG13137_3' side_(2,1)	chr2L:10176091-10176092	CG13137	FBgn0032188
Lrr47	1	Lrr47_3' side_(3,51)	chr2L:10520911-10520912	CG6098	FBgn0010398
CG14926	1	CG14926_3' side_(3,4)	chr2L:11337141-11337142	CG14926	FBgn0032360
CG15483	1	CG15483_3' side_(2,44)	chr2L:12750611-12750612	CG15483	FBgn0032457
CG42680	1	CG42680_3' side_(2,47)	chr2L:14579051-14579052	CG42680	FBgn0261566
stc	1	stc_3' side_(2,83)	chr2L:15118551-15118552	CG3647	FBgn0001978
wor	1	wor_3' side_(4,02)	chr2L:15412371-15412372	CG4158	FBgn0001983
Tim17b2	1	Tim17b2_3' side_(2,03)	chr2L:15490611-15490612	CG15257	FBgn0020371
CG31804	1	CG31804_3' side_(5,39)	chr2L:17366331-17366332	CG31804	FBgn0051804
CG34007	1	CG34007_3' side_(2,44)	chr2L:20496531-20496532	CG34007	FBgn0054007
tsh	1	tsh_3' side_(2,78)	chr2L:21851591-21851592	CG1374	FBgn0003866
CG43750	1	CG43750_3' side_(3,71)	chr2L:2388751-2388752	CG43750	FBgn0264081
slp2	1	slp2_3' side_(4,37)	chr2L:3846571-3846572	CG2939	FBgn0004567
GluRIIB	1	GluRIIB_3' side_(2,1)	chr2L:5565331-5565332	CG7234	FBgn0020429
Osm6	1	Osm6_3' side_(2,55)	chr2L:6555351-6555352	CG9595	FBgn0031829
hui	1	hui_3' side_(3,18)	chr2R:10701571-10701572	CG10200	FBgn0033968
NaPi-T	1	NaPi-T_3' side_(2,74)	chr2R:10729541-10729542	CG10207	FBgn0016684
CG8405	1	CG8405_3' side_(2,97)	chr2R:11954991-11954992	CG8405	FBgn0034071
Or56a	1	Or56a_3' side_(5,7)	chr2R:15656401-15656402	CG12501	FBgn0034473
Sfp60F	1	Sfp60F_3' side_(2,19)	chr2R:21058431-21058432	CG42478	FBgn0259968
eve	1	eve_3' side_(4,38)	chr2R:5869441-5869442	CG2328	FBgn0000606
CG8298	1	CG8298_3' side_(2,15)	chr2R:7880391-7880392	CG8298	FBgn0033673
CG13186	1	CG13186_3' side_(2,1)	chr2R:7970331-7970332	CG13186	FBgn0033680
CG13324	1	CG13324_3' side_(2,74)	chr2R:8936231-8936232	CG13324	FBgn0033789
CG17048	1	CG17048_3' side_(2,41)	chr2R:9215451-9215452	CG17048	FBgn0033828
caup	1	caup_3' side_(4,17)	chr3L:12633131-12633132	CG10605	FBgn0015919
CG33262	1	CG33262_3' side_(2,65)	chr3L:13131041-13131042	CG33262	FBgn0053262
CG42758	1	CG42758_3' side_(5,75)	chr3L:14562781-14562782	CG42758	FBgn0261816
ND-24L	1	ND-24L_3' side_(2,96)	chr3L:17311271-17311272	CG6485	FBgn0036706
tap	1	tap_3' side_(2,11)	chr3L:17356351-17356352	CG7659	FBgn0015550
CG13891	1	CG13891_3' side_(3,15)	chr3L:429131-429132	CG13891	FBgn0035139
CG18586	1	CG18586_3' side_(2,96)	chr3L:5870331-5870332	CG18586	FBgn0035642
DNApol-epsilon58	1	DNApol-epsilon58_3' side_(3,01)	chr3L:5886111-5886112	CG10489	FBgn0035644
vvl	1	vvl_3' side_(3,45)	chr3L:6829811-6829812	CG10037	FBgn0086680
Prat2	1	Prat2_3' side_(3,72)	chr3L:6878461-6878462	CG10078	FBgn0041194
exex	1	exex_3' side_(3,95)	chr3L:7948231-7948232	CG8254	FBgn0041156
Abd-B	1	Abd-B_3' side_(2,24)	chr3R:12719251-12719252	CG11648	FBgn0000015
slou	1	slou_3' side_(4,32)	chr3R:17371381-17371382	CG6534	FBgn0002941
CG42789	1	CG42789_3' side_(2)	chr3R:22602251-22602252	CG42789	FBgn0261860
fkh	1	fkh_3' side_(2,13)	chr3R:24399431-24399432	CG10002	FBgn0000659
zen	1	zen_3' side_(2,55)	chr3R:2577851-2577852	CG1046	FBgn0004053
CecC	1	CecC_3' side_(4,15)	chr3R:26042831-26042832	CG1373	FBgn0000279
CG2053	1	CG2053_3' side_(2,25)	chr3R:27866201-27866202	CG2053	FBgn0039887
CG14598	1	CG14598_3' side_(2,94)	chr3R:3272431-3272432	CG14598	FBgn0037503
CG18249	1	CG18249_3' side_(3,1)	chr3R:4051371-4051372	CG18249	FBgn0037553
CG14662	1	CG14662_3' side_(2,79)	chr3R:803981-803982	CG14662	FBgn0037291
grsm	1	grsm_3' side_(3,02)	chr3R:8771151-8771152	CG7340	FBgn0040493
CG8141	1	CG8141_3' side_(2,78)	chr3R:9026671-9026672	CG8141	FBgn0038125
Art9	1	Art9_3' side_(5,66)	chr3R:9756151-9756152	CG9929	FBgn0038188
CG9926	1	CG9926_3' side_(1,98)	chr3R:9775571-9775572	CG9926	FBgn0038190
CG11448	1	CG11448_3' side_(3)	chrX:1291891-1291892	CG11448	FBgn0024985
disco	1	disco_3' side_(2,05)	chrX:16102761-16102762	CG9908	FBgn0000459
CG12679	1	CG12679_3' side_(5,12)	chrX:20231811-20231812	CG12679	FBgn0031103
CG1324	1	CG1324_3' side_(3,53)	chrX:20594231-20594232	CG1324	FBgn0031129
CG6379	1	CG6379_3' side_(2,47)	chrX:3989111-3989112	CG6379	FBgn0029693
CG43135	1	CG43135_3' side_(3,64)	chrX:4143611-4143612	CG43135	FBgn0262609
CG15577	1	CG15577_3' side_(5,39)	chrX:4168311-4168312	CG15577	FBgn0040904
rux	1	rux_3' side_(3,88)	chrX:5926071-5926072	CG4336	FBgn0003302
oc	1	oc_3' side_(4,07)**	chrX:8511071-8511072	CG12154	FBgn0004102
CG1791	1	CG1791_3' side_(2,61)	chrX:9854911-9854912	CG1791	FBgn0030163
Osi21	1	Osi21_5' side_(8,39)	chr2L:11297641-11297642	CG14925	FBgn0032359
sala	1	sala_5' side_(2,44)	chr2L:11473331-11473332	CG4922	FBgn0003313
robo3	1	robo3_5' side_(3,81)	chr2L:1252221-1252222	CG5423	FBgn0041097
CG16800	1	CG16800_5' side_(2,41)	chr2L:12949641-12949642	CG16800	FBgn0032462
noc	1	noc_5' side_(2,12)	chr2L:14482821-14482822	CG4491	FBgn0005771

ProtB	1	ProtB_5' side_(2,65)	chr2L:14898851-14898852	CG4478	FBgn0013301
CaBP1	1	CaBP1_5' side_(4,13)	chr2L:16358211-16358212	CG5809	FBgn0025678
ApepP	1	ApepP_5' side_(2,69)	chr2L:16919431-16919432	CG6291	FBgn0026150
aop	1	aop_5' side_(2,01)	chr2L:2182161-2182162	CG3166	FBgn0000097
CG3557	1	CG3557_5' side_(2,1)	chr2L:2387031-2387032	CG3557	FBgn0031423
CG2955	1	CG2955_5' side_(6,24)	chr2L:4136931-4136932	CG2955	FBgn0031585
CG14020	1	CG14020_5' side_(2,32)	chr2L:5515101-5515102	CG14020	FBgn0031707
CG31639	1	CG31639_5' side_(3,51)	chr2L:6155731-6155732	CG31639	FBgn0051639
Ugt37b1	1	Ugt37b1_5' side_(2,51)	chr2L:6216141-6216142	CG9481	FBgn0026755
CG17375	1	CG17375_5' side_(2,51)	chr2L:6810511-6810512	CG17375	FBgn0031861
raw	1	raw_5' side_(6,09)	chr2L:8746201-8746202	CG12437	FBgn0003209
CG12438	1	CG12438_5' side_(3,17)	chr2L:8746811-8746812	CG12438	FBgn0032065
Lsp1beta	1	Lsp1beta_5' side_(2,19)	chr2L:892871-892872	CG4178	FBgn0002563
bib	1	bib_5' side_(3,07)	chr2L:9981751-9981752	CG4722	FBgn0000180
CG12862	1	CG12862_5' side_(3,34)	chr2R:10528601-10528602	CG12862	FBgn0033950
CG30083	1	CG30083_5' side_(3,39)	chr2R:11604371-11604372	CG30083	FBgn0050083
CG17290	1	CG17290_5' side_(3,64)	chr2R:13040951-13040952	CG17290	FBgn0034201
Idgf5	1	Idgf5_5' side_(3,99)	chr2R:14280601-14280602	CG5154	FBgn0064237
CG42306	1	CG42306_5' side_(2,37)	chr2R:14550971-14550972	CG42306	FBgn0259202
Obp56g	1	Obp56g_5' side_(2,95)	chr2R:15674511-15674512	CG13873	FBgn0034474
Obp56h	1	Obp56h_5' side_(2,01)	chr2R:15690761-15690762	CG13874	FBgn0034475
CG13872	1	CG13872_5' side_(2,82)	chr2R:15777731-15777732	CG13872	FBgn0034477
CG12484	1	CG12484_5' side_(4,73)	chr2R:16301171-16301172	CG12484	FBgn0086604
HmgZ	1	HmgZ_5' side_(2,24)	chr2R:17594791-17594792	CG17921	FBgn0010228
CG10384	1	CG10384_5' side_(2,94)	chr2R:18349101-18349102	CG10384	FBgn0034731
CG3092	1	CG3092_5' side_(3,35)	chr2R:19115011-19115012	CG3092	FBgn0034835
nvy	1	nvy_5' side_(2,55)	chr2R:20162491-20162492	CG3385	FBgn0005636
CG30430	1	CG30430_5' side_(3,56)	chr2R:21007291-21007292	CG30430	FBgn0050430
SdhB	1	SdhB_5' side_(4,1)	chr2R:2693151-2693152	CG3283	FBgn0014028
stmA	1	stmA_5' side_(2,03)	chr2R:4621901-4621902	CG8739	FBgn0086784
sns	1	sns_5' side_(3,21)	chr2R:4684751-4684752	CG33141	FBgn0024189
Hdc	1	Hdc_5' side_(3,84)	chr2R:6126571-6126572	CG3454	FBgn0005619
Cyp12d1-d	1	Cyp12d1-d_5' side_(2,66)	chr2R:7017791-7017792	CG33503	FBgn0053503
TpnC47D	1	TpnC47D_5' side_(2,87)	chr2R:7162831-7162832	CG9073	FBgn0010423
wuc	1	wuc_5' side_(2,52)	chr2R:8586911-8586912	CG12442	FBgn0033770
CG42663	1	CG42663_5' side_(2,48)*	chr2R:8589511-8589512	CG42663	FBgn0261545
CG41242	1	CG41242_5' side_(1,98)	chr2RHET:2158471-2158472	CG41242	FBgn0085569
CG12523	1	CG12523_5' side_(2,82)	chr3L:10790891-10790892	CG12523	FBgn0036102
CG32105	1	CG32105_5' side_(2,14)	chr3L:12315161-12315162	CG32105	FBgn0052105
caps	1	caps_5' side_(3,5)	chr3L:13203031-13203032	CG11282	FBgn0023095
comm	1	comm_5' side_(2,24)	chr3L:15755581-15755582	CG17943	FBgn0010105
CG42852	1	CG42852_5' side_(3,29)	chr3L:16741881-16741882	CG42852	FBgn0262099
grim	1	grim_5' side_(2,15)	chr3L:18316161-18316162	CG4345	FBgn0015946
CG43407	1	CG43407_5' side_(2,44)	chr3L:19050671-19050672	CG43407	FBgn0263326
CG43312	1	CG43312_5' side_(2,71)	chr3L:22486031-22486032	CG43312	FBgn0263004
CG14985	1	CG14985_5' side_(3,38)	chr3L:4000371-4000372	CG14985	FBgn0035482
CG10479	1	CG10479_5' side_(2,03)*	chr3L:5981271-5981272	CG10479	FBgn0035656
CG13293	1	CG13293_5' side_(5,83)	chr3L:6075811-6075812	CG13293	FBgn0035677
CG7546	1	CG7546_5' side_(2,25)	chr3L:7503391-7503392	CG7546	FBgn0035793
hng3	1	hng3_5' side_(2,35)	chr3L:776971-776972	CG13897	FBgn0035160
bip1	1	bip1_5' side_(2,72)	chr3L:8053031-8053032	CG7574	FBgn0026263
CG13678	1	CG13678_5' side_(3,3)	chr3L:8213711-8213712	CG13678	FBgn0035859
CG5194	1	CG5194_5' side_(3,34)	chr3L:9004911-9004912	CG5194	FBgn0035955
nerfin-1	1	nerfin-1_5' side_(5,59)	chr3L:907761-907762	CG13906	FBgn0028999
AOX4	1	AOX4_5' side_(2,66)	chr3R:11386051-11386052	CG18516	FBgn0038350
pnr	1	pnr_5' side_(3,35)	chr3R:11851551-11851552	CG3978	FBgn0003117
Glut3	1	Glut3_5' side_(2,37)	chr3R:12606731-12606732	CG3853	FBgn0015230
CG7587	1	CG7587_5' side_(1,97)	chr3R:13415591-13415592	CG7587	FBgn0038523
gl	1	gl_5' side_(2,55)	chr3R:14202611-14202612	CG7672	FBgn0004618
cas	1	cas_5' side_(2,2)	chr3R:1548911-1548912	CG2102	FBgn0004878
Rh3	1	Rh3_5' side_(2,7)	chr3R:15910051-15910052	CG10888	FBgn0003249
CG11373	1	CG11373_5' side_(2,02)	chr3R:1788711-1788712	CG11373	FBgn0040679
CG11459	1	CG11459_5' side_(3,13)	chr3R:1857471-1857472	CG11459	FBgn0037396
E(spl)malpha-BFM	1	E(spl)malpha-BFM_5' side_(2,67)	chr3R:21835771-21835772	CG8337	FBgn0002732
CG31324	1	CG31324_5' side_(3,32)	chr3R:21932091-21932092	CG31324	FBgn0051324
Tl	1	Tl_5' side_(4)	chr3R:22623611-22623612	CG5490	FBgn0262473
unc80	1	unc80_5' side_(2,34)	chr3R:23487411-23487412	CG18437	FBgn0039536
CG34354	1	CG34354_5' side_(2,75)	chr3R:23934791-23934792	CG34354	FBgn0085383
Dr	1	Dr_5' side_(3,53)	chr3R:25378681-25378682	CG1897	FBgn0000492
Ama	1	Ama_5' side_(2,53)	chr3R:2587401-2587402	CG2198	FBgn0000071
CG1340	1	CG1340_5' side_(3,15)	chr3R:26544151-26544152	CG1340	FBgn0039797
CG11741	1	CG11741_5' side_(2,18)	chr3R:4354971-4354972	CG11741	FBgn0040531
beat-Va	1	beat-Va_5' side_(2,13)	chr3R:8674371-8674372	CG10134	FBgn0038087
CG43131	1	CG43131_5' side_(3,77)	chr3R:939431-939432	CG43131	FBgn0262605
ems	1	ems_5' side_(2,52)	chr3R:9723431-9723432	CG2988	FBgn0000576
dpr16	1	dpr16_5' side_(4,72)	chr3R:976391-976392	CG12591	FBgn0037295

fd102C	1	fd102C_5' side_(2,51)	chr4:871071-871072	CG11152	FBgn0039937
CG42300	1	CG42300_5' side_(2,21)	chrX:15454071-15454072	CG42300	FBgn0259196
unc-4	1	unc-4_5' side_(2,32)	chrX:17651841-17651842	CG6269	FBgn0024184
CG7423	1	CG7423_5' side_(3,82)	chrX:18981811-18981812	CG7423	FBgn0030982
CG12688	1	CG12688_5' side_(4,76)	chrX:4270381-4270382	CG12688	FBgn0029707
CG42749	1	CG42749_5' side_(3,61)	chrX:5163591-5163592	CG42749	FBgn0261803
CG1958	1	CG1958_5' side_(2,08)	chrX:7150701-7150702	CG1958	FBgn0029940
CG1402	1	CG1402_5' side_(4,23)	chrX:7702311-7702312	CG1402	FBgn0029962
CG7065	1	CG7065_5' side_(5,14)	chrX:9035651-9035652	CG7065	FBgn0030091
bsk	1	bsk_Intragenic_(2,18)	chr2L:10249431-10249432	CG5680	FBgn0000229
CG34367	1	CG34367_Intragenic_(4,57)	chr2L:10349751-10349752	CG34367	FBgn0085396
Myo31DF	1	Myo31DF_Intragenic_(2,27)	chr2L:10503591-10503592	CG7438	FBgn0086347
Pde1c	1	Pde1c_Intragenic_(2,65)	chr2L:11895151-11895152	CG44007	FBgn0264815
bun	1	bun_Intragenic_(3)	chr2L:12529681-12529682	CG42281	FBgn0259176
kuz	1	kuz_Intragenic_(2,96)	chr2L:13576411-13576412	CG7147	FBgn0259984
cenG1A	1	cenG1A_Intragenic_(4,6)	chr2L:13877171-13877172	CG31811	FBgn0028509
osp	1	osp_Intragenic_(2,59)	chr2L:14683811-14683812	CG3479	FBgn0003016
crp	1	crp_Intragenic_(1,94)	chr2L:16275391-16275392	CG7664	FBgn0001994
CG42389	1	CG42389_Intragenic_(1,96)	chr2L:16647531-16647532	CG42389	FBgn0259735
beat-IIIa	1	beat-IIIa_Intragenic_(2,07)	chr2L:17163991-17163992	CG12621	FBgn0265607
beat-IIIc	1	beat-IIIc_Intragenic_(2,66)	chr2L:17221181-17221182	CG15138	FBgn0032629
Dhc36C	1	Dhc36C_Intragenic_(4,17)	chr2L:17514751-17514752	CG5526	FBgn0013810
kon	1	kon_Intragenic_(3,38)	chr2L:18498891-18498892	CG10275	FBgn0032683
ssp3	1	ssp3_Intragenic_(4,14)	chr2L:18925451-18925452	CG18397	FBgn0032723
brat	1	brat_Intragenic_(2,38)	chr2L:19167641-19167642	CG10719	FBgn0010300
dnt	1	dnt_Intragenic_(2,38)	chr2L:19341711-19341712	CG17559	FBgn0024245
erm	1	erm_Intragenic_(4,85)	chr2L:1959431-1959432	CG31670	FBgn0031375
cad	1	cad_Intragenic_(3,07)	chr2L:20778501-20778502	CG1759	FBgn0000251
CG9328	1	CG9328_Intragenic_(7,85)	chr2L:20800351-20800352	CG9328	FBgn0032886
CG3645	1	CG3645_Intragenic_(4,46)	chr2L:268371-268372	CG3645	FBgn0031238
Plc21C	1	Plc21C_Intragenic_(2,48)	chr2L:306591-306592	CG4574	FBgn0004611
toc	1	toc_Intragenic_(1,96)	chr2L:3117091-3117092	CG9660	FBgn0015600
fred	1	fred_Intragenic_(2,38)	chr2L:3939351-3939352	CG31774	FBgn0051774
tkv	1	tkv_Intragenic_(4,24)	chr2L:5245591-5245592	CG14026	FBgn0003716
ush	1	ush_Intragenic_(2,52)	chr2L:526161-526162	CG2762	FBgn0003963
DIP-eta	1	DIP-eta_Intragenic_(2,58)	chr2L:5612131-5612132	CG14010	FBgn0031725
Gsc	1	Gsc_Intragenic_(4,29)	chr2L:589251-589252	CG2851	FBgn0010323
stai	1	stai_Intragenic_(2,5)	chr2L:6104401-6104402	CG31641	FBgn0266521
PDZ-GEF	1	PDZ-GEF_Intragenic_(3,17)	chr2L:6324531-6324532	CG9491	FBgn0265778
sens-2	1	sens-2_Intragenic_(2,17)	chr2L:6828741-6828742	CG31632	FBgn0051632
Wnt4	1	Wnt4_Intragenic_(3,04)	chr2L:7272801-7272802	CG4698	FBgn0010453
CG14535	1	CG14535_Intragenic_(5,06)	chr2L:7850661-7850662	CG14535	FBgn0031955
fu12	1	fu12_Intragenic_(2,65)	chr2L:8463491-8463492	CG17608	FBgn0026718
Sema-1a	1	Sema-1a_Intragenic_(2,37)	chr2L:8578571-8578572	CG18405	FBgn0011259
numb	1	numb_Intragenic_(3,72)	chr2L:9443381-9443382	CG3779	FBgn0002973
Shroom	1	Shroom_Intragenic_(3,54)	chr2R:10221881-10221882	CG34379	FBgn0085408
L	1	L_Intragenic_(1,97)	chr2R:10369501-10369502	-	FBgn0267825
mspo	1	mspo_Intragenic_(2,85)	chr2R:10592791-10592792	CG10145	FBgn0020269
Hr51	1	Hr51_Intragenic_(2,79)	chr2R:11222291-11222292	CG16801	FBgn0034012
ns2	1	ns2_Intragenic_(2,85)	chr2R:13455731-13455732	CG6501	FBgn0034243
sm	1	sm_Intragenic_(2,3)	chr2R:15447831-15447832	CG9218	FBgn0003435
Fili	1	Fili_Intragenic_(4,84)	chr2R:17779111-17779112	CG34368	FBgn0085397
Liprin-gamma	1	Liprin-gamma_Intragenic_(2,38)	chr2R:18252791-18252792	CG11206	FBgn0034720
uzip	1	uzip_Intragenic_(3,79)	chr2R:20900551-20900552	CG3533	FBgn0004055
esn	1	esn_Intragenic_(2,84)	chr2R:2985051-2985052	CG43722	FBgn0263934
so	1	so_Intragenic_(5,32)	chr2R:3320731-3320732	CG11121	FBgn0003460
CG1399	1	CG1399_Intragenic_(3,3)	chr2R:3585351-3585352	CG1399	FBgn0033212
LRP1	1	LRP1_Intragenic_(4,7)	chr2R:4082651-4082652	CG33087	FBgn0053087
pdm3	1	pdm3_Intragenic_(3,62)	chr2R:4270831-4270832	CG42698	FBgn0261588
Cyp4ad1	1	Cyp4ad1_Intragenic_(2,03)	chr2R:4329811-4329812	CG2110	FBgn0033292
CAP	1	CAP_Intragenic_(7,08)	chr2R:6157291-6157292	CG18408	FBgn0033504
inv	1	inv_Intragenic_(5,67)	chr2R:7365531-7365532	CG17835	FBgn0001269
Psc	1	Psc_Intragenic_(2,44)	chr2R:8861251-8861252	CG3886	FBgn0005624
Drl-2	1	Drl-2_Intragenic_(3,18)	chr2R:8954951-8954952	CG3915	FBgn0033791
Ack-like	1	Ack-like_Intragenic_(2,25)	chr2R:9039071-9039072	CG43741	FBgn0263998
arr	1	arr_Intragenic_(2,47)	chr2R:9362251-9362252	CG5912	FBgn0000119
Vmat	1	Vmat_Intragenic_(2,28)	chr2R:9415231-9415232	CG33528	FBgn0260964
mam	1	mam_Intragenic_(2,21)	chr2R:9915851-9915852	CG8118	FBgn0002643
dpr6	1	dpr6_Intragenic_(2,09)	chr3L:10001831-10001832	CG14162	FBgn0040823
simj	1	simj_Intragenic_(1,96)	chr3L:10676471-10676472	CG32067	FBgn0010762
CG32085	1	CG32085_Intragenic_(2,78)	chr3L:11664881-11664882	CG32085	FBgn0052085
CG11658	1	CG11658_Intragenic_(2,82)	chr3L:11701611-11701612	CG11658	FBgn0036196
Nrx-IV	1	Nrx-IV_Intragenic_(4,11)	chr3L:12140461-12140462	CG6827	FBgn0013997
sowah	1	sowah_Intragenic_(2,41)	chr3L:12556111-12556112	CG10632	FBgn0036302
Ptp61F	1	Ptp61F_Intragenic_(2,75)	chr3L:1390451-1390452	CG9181	FBgn0267487
Sox21b	1	Sox21b_Intragenic_(2,59)	chr3L:14107611-14107612	CG32139	FBgn0042630

HGTX	1	HGTX_Intragenic_(3,71)	chr3L:14584521-14584522	CG13475	FBgn0040318
mnd	1	mnd_Intragenic_(2,12)	chr3L:14981271-14981272	CG3297	FBgn0002778
Abl	1	Abl_Intragenic_(2,48)	chr3L:16634421-16634422	CG4032	FBgn0000017
CG9701	1	CG9701_Intragenic_(2,67)	chr3L:16731841-16731842	CG9701	FBgn0036659
Nrt	1	Nrt_Intragenic_(2,61)	chr3L:16763851-16763852	CG9704	FBgn0004108
Rbp6	1	Rbp6_Intragenic_(2,44)	chr3L:17106671-17106672	CG32169	FBgn0260943
nkd	1	nkd_Intragenic_(2,11)	chr3L:19018931-19018932	CG11614	FBgn0002945
Dbx	1	Dbx_Intragenic_(2,29)	chr3L:1931511-1931512	CG42234	FBgn0261723
CG42674	1	CG42674_Intragenic_(2,24)	chr3L:20159671-20159672	CG42674	FBgn0261556
gogo	1	gogo_Intragenic_(2,41)	chr3L:20270111-20270112	CG32227	FBgn0052227
CG10508	1	CG10508_Intragenic_(3,59)	chr3L:21204931-21204932	CG10508	FBgn0037060
Mrtf	1	Mrtf_Intragenic_(2,57)	chr3L:2745211-2745212	CG32296	FBgn0052296
Tet	1	Tet_Intragenic_(2,97)	chr3L:2809691-2809692	CG43444	FBgn0263392
Eip63E	1	Eip63E_Intragenic_(2,88)	chr3L:3544051-3544052	CG10579	FBgn0005640
trh	1	trh_Intragenic_(2,37)	chr3L:378311-378312	CG42865	FBgn0262139
Gad1	1	Gad1_Intragenic_(3,96)	chr3L:4077171-4077172	CG14994	FBgn0004516
CG14989	1	CG14989_Intragenic_(5,7)	chr3L:4088911-4088912	CG14989	FBgn0035495
Cip4	1	Cip4_Intragenic_(2,14)	chr3L:4357151-4357152	CG15015	FBgn0035533
Src64B	1	Src64B_Intragenic_(2,44)	chr3L:4617321-4617322	CG7524	FBgn0262733
Con	1	Con_Intragenic_(2,03)	chr3L:4987131-4987132	CG7503	FBgn0005775
Cyt-c1	1	Cyt-c1_Intragenic_(2,02)	chr3L:5353951-5353952	CG4769	FBgn0035600
vn	1	vn_Intragenic_(2,77)	chr3L:5831521-5831522	CG10491	FBgn0003984
CG33275	1	CG33275_Intragenic_(2,77)	chr3L:7627871-7627872	CG33275	FBgn0035802
CG32369	1	CG32369_Intragenic_(2,58)	chr3L:7796831-7796832	CG32369	FBgn0052369
Gug	1	Gug_Intragenic_(2,31)	chr3L:8453671-8453672	CG6964	FBgn0010825
dally	1	dally_Intragenic_(2,77)	chr3L:8847871-8847872	CG4974	FBgn0263930
CG6767	1	CG6767_Intragenic_(3,51)	chr3L:9687751-9687752	CG6767	FBgn0036030
cno	1	cno_Intragenic_(2,07)	chr3R:1014761-1014762	CG42312	FBgn0259212
SF2	1	SF2_Intragenic_(2,18)	chr3R:12167691-12167692	CG6987	FBgn0040284
Fas1	1	Fas1_Intragenic_(8,84)	chr3R:12455681-12455682	CG6588	FBgn0262742
osa	1	osa_Intragenic_(2,31)	chr3R:13537331-13537332	CG7467	FBgn0261885
Ssdp	1	Ssdp_Intragenic_(2,24)	chr3R:14021851-14021852	CG7187	FBgn0011481
14-3-3epsilon	1	14-3-3epsilon_Intragenic_(3,16)	chr3R:14071471-14071472	CG31196	FBgn0020238
Cha	1	Cha_Intragenic_(3,3)	chr3R:14550651-14550652	CG12345	FBgn0000303
gukh	1	gukh_Intragenic_(2,61)	chr3R:14821951-14821952	CG31043	FBgn0026239
cdi	1	cdi_Intragenic_(2,5)	chr3R:14906791-14906792	CG6027	FBgn0004876
Dl	1	Dl_Intragenic_(6,94)	chr3R:15145131-15145132	CG3619	FBgn0000463
Dys	1	Dys_Intragenic_(2,2)	chr3R:15311871-15311872	CG34157	FBgn0260003
CG2082	1	CG2082_Intragenic_(2,32)	chr3R:1599601-1599602	CG2082	FBgn0027608
Calx	1	Calx_Intragenic_(2,57)	chr3R:16821991-16821992	CG5685	FBgn0013995
Rm62	1	Rm62_Intragenic_(2,35)	chr3R:1832551-1832552	CG10279	FBgn0003261
wge	1	wge_Intragenic_(7,87)	chr3R:18545291-18545292	CG31151	FBgn0051151
cnc	1	cnc_Intragenic_(8,03)	chr3R:19037651-19037652	CG43286	FBgn0262975
pnt	1	pnt_Intragenic_(2,38)	chr3R:19164291-19164292	CG17077	FBgn0003118
CG4393	1	CG4393_Intragenic_(1,96)	chr3R:19209671-19209672	CG4393	FBgn0039075
tnc	1	tnc_Intragenic_(4,36)	chr3R:20826131-20826132	CG13648	FBgn0039257
Fur1	1	Fur1_Intragenic_(4,16)	chr3R:21189711-21189712	CG10772	FBgn0004509
gpp	1	gpp_Intragenic_(3,59)	chr3R:2253691-2253692	CG42803	FBgn0264495
Tusp	1	Tusp_Intragenic_(2,17)	chr3R:23442561-23442562	CG5586	FBgn0039530
CG34353	1	CG34353_Intragenic_(2,26)	chr3R:23662061-23662062	CG34353	FBgn0085382
CG34362	1	CG34362_Intragenic_(5,56)	chr3R:23887651-23887652	CG34362	FBgn0085391
CG11898	1	CG11898_Intragenic_(1,98)	chr3R:24995231-24995232	CG11898	FBgn0039645
pb	1	pb_Intragenic_(2,65)	chr3R:2561951-2561952	CG31481	FBgn0051481
Dfd	1	Dfd_Intragenic_(2,07)	chr3R:2624911-2624912	CG2189	FBgn0000439
zfh1	1	zfh1_Intragenic_(4,02)	chr3R:26603991-26603992	CG1322	FBgn0004606
wts	1	wts_Intragenic_(4,14)	chr3R:26629031-26629032	CG12072	FBgn0011739
cindr	1	cindr_Intragenic_(2,67)	chr3R:26643031-26643032	CG31012	FBgn0027598
CG34347	1	CG34347_Intragenic_(2,64)	chr3R:27176451-27176452	CG34347	FBgn0085376
Poxm	1	Poxm_Intragenic_(3,08)	chr3R:4156681-4156682	CG9610	FBgn0003129
CG34114	1	CG34114_Intragenic_(2,18)	chr3R:6857651-6857652	CG34114	FBgn0083950
pros	1	pros_Intragenic_(3,26)	chr3R:7198641-7198642	CG17228	FBgn0004595
Jupiter	1	Jupiter_Intragenic_(2,35)	chr3R:7441281-7441282	CG31363	FBgn0051363
beat-Vc	1	beat-Vc_Intragenic_(2,24)	chr3R:8581931-8581932	CG14390	FBgn0038084
sim	1	sim_Intragenic_(2,08)	chr3R:8891111-8891112	CG7771	FBgn0004666
corto	1	corto_Intragenic_(2,42)	chr3R:908511-908512	CG2530	FBgn0010313
sv	1	sv_Intragenic_(5,31)	chr4:1125481-1125482	CG11049	FBgn0005561
fuss	1	fuss_Intragenic_(2,38)	chr4:994311-994312	CG11093	FBgn0039932
klhl10	1	klhl10_Intragenic_(2,15)	chrU:1932421-1932422	CG12423	FBgn0040038
CG45781	1	CG45781_Intragenic_(2,16)	chrU:80571-80572	CG45781	FBgn0267428
Tis11	1	Tis11_Intragenic_(2,51)	chrX:12562711-12562712	CG4070	FBgn0011837
Smr	1	Smr_Intragenic_(9,38)	chrX:12630311-12630312	CG4013	FBgn0265523
NetB	1	NetB_Intragenic_(2,87)	chrX:14619161-14619162	CG10521	FBgn0015774
acj6	1	acj6_Intragenic_(3,56)	chrX:15259171-15259172	CG9151	FBgn0000028
Sep4	1	Sep4_Intragenic_(10,58)	chrX:16583781-16583782	CG9699	FBgn0259923
Bx	1	Bx_Intragenic_(2,44)	chrX:18442611-18442612	CG44425	FBgn0265598
Rip11	1	Rip11_Intragenic_(3,03)	chrX:18537151-18537152	CG6606	FBgn0027335

kek5	1	kek5_Intragenic_(2,38)	chrX:19266171-19266172	CG12199	FBgn0031016
RhoGAP19D	1	RhoGAP19D_Intragenic_(4,51)	chrX:20370331-20370332	CG1412	FBgn0031118
phl	1	phl_Intragenic_(6,51)	chrX:2215111-2215112	CG2845	FBgn0003079
dnc	1	dnc_Intragenic_(3,41)	chrX:3103351-3103352	CG32498	FBgn0000479
CG4293	1	CG4293_Intragenic_(3,1)	chrX:424211-424212	CG4293	FBgn0024983
CG3062	1	CG3062_Intragenic_(3,64)	chrX:4537551-4537552	CG3062	FBgn0025612
CG2861	1	CG2861_Intragenic_(2,89)	chrX:4692171-4692172	CG2861	FBgn0029728
Vsx1	1	Vsx1_Intragenic_(3,62)	chrX:5504511-5504512	CG4136	FBgn0263511
IntS6	1	IntS6_Intragenic_(2,23)	chrX:5638971-5638972	CG3125	FBgn0261383
Grip	1	Grip_Intragenic_(5,93)	chrX:5863711-5863712	CG14447	FBgn0029830
pigs	1	pigs_Intragenic_(2,69)	chrX:6495591-6495592	CG3973	FBgn0029881
CG12541	1	CG12541_Intragenic_(7,9)	chrX:6941051-6941052	CG12541	FBgn0029930
CG43867	1	CG43867_Intragenic_(5,95)	chrX:736391-736392	CG43867	FBgn0264449
Lim1	1	Lim1_Intragenic_(5,34)	chrX:8663611-8663612	CG11354	FBgn0026411
fend	1	fend_Intragenic_(4,88)	chrX:9025911-9025912	CG12664	FBgn0030090
nub/pdm2	1	nub/pdm2*#_Intragenic_(3,17)	chr2L:12613231-12613232	CG34395/CG12287	FBgn0085424/FBgn0004394
ITP/CG4622	1	ITP/CG4622_Intragenic_(3,14)	chr2R:20432181-20432182	CG13586/CG4622	FBgn0035023/FBgn0035021
kermit/Lpin	1	kermit/Lpin_Intragenic_(2,2)	chr2R:4035411-4035412	CG11546/CG8709	FBgn0010504/FBgn0263593
Cyp49a1/G-oalpha47A	1	Cyp49a1/G-oalpha47A_Intragenic_(2,83)	chr2R:6344061-6344062	CG18377/CG2204	FBgn0033524/FBgn0001122
CG43954/Lasp	1	CG43954/Lasp_Intragenic_(2,64)	chr3L:16694531-16694532	CG43954/CG3849	FBgn0264605/FBgn0063485
Cha/VAChT	1	Cha/VAChT_Intragenic_(2,06)	chr3R:14534501-14534502	CG12345/CG32848	FBgn0000302/FBgn0270928
