# Supplementary material for: Genome-Wide Mapping of Collier In Vivo Binding Sites Highlights Its Hierarchical Position in Different Transcription Regulatory Networks
Source: PLoS One. 2015 Jul 23;10(7):e0133387. doi: 10.1371/journal.pone.0133387 (PMC4512700; doi:10.1371/journal.pone.0133387)
Supplement: S2 Table — (PDF) [file pone.0133387.s010.pdf]

Table S2. Genomic coordinates and annotation of candidate Col-regulated CRM

Curated annotation of the Col binding site

Col target gene	Open chromatin marks	Mef-2 binding site<0.5 kb	Reporter Name	Previously characterized CRMs http://redfly.ccr.buffalo.edu/	Genomic coordinates (size)	MEME Col binding site **mutated positions** *polymorphism
Ama	+		*Ama*_Col		Chr3R: 2.587.010-2.587.909 (**899**)	CCT**CCC**ATGGGAACG
ap			*ap*_Col		Chr2R: 1.623.820-1.625.439 (**1619**)	ACT**CCC**TTCGGAATA --// 603pb //-- GAA**CCC**AAGGGACGA
cnc			*cnc*_Col		Chr3R: 19.037.100-19.038.139 (**1039**)	AATTCt*CCA**GGG**ACC
eya	+		*eya*_Col		Chr2L: 6.540.104-6.541.545 (**1441**)	AGAC**CCC**CAGGGATG --// 375pb //-- GCA**CCC**CAGGGCTTA
jing	+		*jing*_Col		Chr2R: 2.482.100-2.483.002 (**903**)	TGCCCGCT**GGG**AATT
Mrtf		+	*Mrtf*_Col		Chr3L: 2.744.715-2.745.692 (**978**)	TCCCCCCT**GGG**AATT
nerfin-1			*nerfin-1*_Col	PMID:19056518 Kuzin et al., 2008	Chr3L: 907324-908228 (**900**)	ATT**CCC**CTGGCAAAA --// 20pb //-- ATCGCCTT**GGG**AATA
Oaz	+		*Oaz*_Col		Chr2R: 10.339.692-10.340.791 (**1099**)	AATCCCAG**GGG**AACC
so	+	+	*so*_Col		Chr2R: 3.320.280 -3.321.219 (**939**)	AATGCCCCG**GGG**AGG
tkv	+	+	*tkv*_Col	PMID:19890324 Zinzen et al., 2009	Chr2L: 5.245.055-5.246.039 (**984**)	GAT**CCC**CAGGGAATT
col	+	+	*col*2.3-0.9	PMID:20056681 Dubois et al., 2010	Chr2R: 10.687.378-10.688.751 (**1373**)	ATGT**CCC**CAGACATC
